# Risk of incident cardiovascular diseases at national and subnational levels in Iran from 2000 to 2016 and projection through 2030: Insights from Iran STEPS surveys

**DOI:** 10.1371/journal.pone.0290006

**Published:** 2023-08-23

**Authors:** Hedyeh Ebrahimi, Masoud Masinaei, Arya Aminorroaya, Zahra Aryan, Parinaz Mehdipour, Yasna Rostam-Abadi, Naser Ahmadi, Sahar Saeedi Moghaddam, Farhad Pishgar, Ali Ghanbari, Nazila Rezaei, Amirhossein Takian, Farshad Farzadfar

**Affiliations:** 1 Non-Communicable Diseases Research Center, Endocrinology and Metabolism Population Sciences Institute, Tehran University of Medical Sciences, Tehran, Iran; 2 Tehran Heart Center, Tehran University of Medical Sciences, Tehran, Iran; 3 Department of Medicine, Rutgers New Jersey Medical School, Newark, New Jersey, United States of America; 4 Centre for Epidemiology and Biostatistics, Melbourne School of Population and Global Health, University of Melbourne, Melbourne, Australia; 5 Iranian National Center for Addiction Studies (INCAS), Tehran University of Medical Sciences, Tehran, Iran; 6 Kiel Institute for the World Economy, Kiel, Germany; 7 Russell H. Morgan Department of Radiology and Radiological Science, Johns Hopkins University, School of Medicine, Baltimore, Maryland, United States of America; 8 Department of Health Management and Economics, School of Public Health, Tehran University of Medical Sciences, Tehran, Iran; 9 Department of Global Health and Public Policy, School of Public Health, Tehran University of Medical Sciences, Tehran, Iran; 10 Health Equity Research Center (HERC), Tehran University of Medical Sciences, Tehran, Iran; 11 Endocrinology and Metabolism Research Center, Endocrinology and Metabolism Clinical Sciences Institute, Tehran University of Medical Sciences, Tehran, Iran; Ochsner Clinic Foundation: Ochsner Health System, UNITED STATES

## Abstract

**Background:**

Cardiovascular Disease (CVD) is the leading cause of death in developing countries. CVD risk stratification guides the health policy to make evidence-based decisions.

**Aim:**

To provide current picture and future trend of CVD risk in the adult Iranian population.

**Methods:**

Nationally representative datasets of 2005, 2006, 2007, 2008, 2009, 2011, and 2016 STEPwise approach to non-communicable diseases risk factor surveillance (STEPS) studies were used to generate the 10-year and 30-year risks of CVD based on Framingham, Globorisk, and World Health Organization (WHO) risk estimation models. Trend of CVD risk was calculated from 2000 until 2016 and projected to 2030.

**Results:**

In 2016, based on Framingham model, 14.0% of the Iranian, aged 30 to 74, were at great risk (≥20%) of CVD in the next 10 years (8.0% among females, 20.7% among males). Among those aged 25 to 59, 12.7% had ≥45% risk of CVD in the coming 30 years (9.2% among females, 16.6 among males). In 2016, CVD risk was higher among urban area inhabitants. Age-standardized Framingham 10-year CVD risk will increase 32.2% and 19%, from 2000 to 2030, in females and males, respectively. Eastern provinces had the lowest and northern provinces had the greatest risk.

**Conclusions:**

This study projected that CVD risk has increased from 2000 to 2016 in Iran. Without further risk factor modification, this trend will continue until 2030. We have identified populations at higher risks of CVD to guide future intervention.

## Introduction

Cardiovascular Disease (CVD) is the leading cause of death in Iran [1]; accounting for almost half of the premature deaths attributed to non-communicable diseases (NCD) [2]. Additionally, the age-standardized incidence rate, age-standardized death rate, and disability-adjusted life years (DALYs) rate of CVD in Iran are higher than the respective global figures [3]. On the other hand, Iran currently has a quite young age structure, with only 6.6% of the total population over 65 years of age in 2020; however, the proportion of the population aged 65 and older will increase to more than 20% in 2050 [4]. Moreover, Iran’s population is moving toward a more sedentary lifestyle, leading to a higher obesity rate and less physical activity in the upcoming years [5]. Considering both population aging and lifestyle changes, the growth in CVD incidence is inevitable in the coming years.

Many guidelines recommend that cardiovascular risk factors should be treated according to the baseline incidence risk of CVD, irrespective of the severity of each risk factor [6–8]. Furthermore, high-risk populations are not usually distributed equally, and healthcare resources are limited in Iran, as well as most other countries. It is also shown that the predictive reliability of CVD risk factors such as LDL-C depends on studied characteristics and its association with CVD incidents can be weak among middle-aged adults [9]. One of the most effective strategies for preventing CVD is to target people at higher risk of CVD events. Hence, effective and efficient resource allocation by health policymakers necessitates delineating the distribution of high-risk populations for CVD events based on geographical location and socio-demographic status. Simplification of CVD risk models without jeopardizing their efficacy in prediction of CVD help providing CVD risk estimates in low resource populations.

Investigating the trend of the risk of incident CVD will provide valuable information about the current drawbacks and present a platform for evaluating the policies in action. Several studies provided distribution of CVD estimates and cardiovascular risk factors individually in Iran [3,10–12]. Nevertheless, the distribution of CVD risk and its trend were not studied systematically at national and subnational levels previously.

In this study, we aimed to describe the estimates of CVD risk at national and subnational levels in Iran from 2005 to 2016 and present a projection from 2000 to 2030. We employed the results of the previous STEPwise approach toward NCD risk factor surveillance (STEPS) surveys in Iran to estimate the CVD risk using Framingham, Globorisk, and World Health Organization (WHO) risk scores.

## Materials and methods

### Data sources

To estimate the CVD risk among the Iranian population, we pooled individual-level data from seven STEPS studies conducted in 2005, 2006, 2007, 2008, 2009, 2011, and 2016. This study follows the GATHER statement guideline to generate the trend of CVD risk in Iran based on the nationally representative population-based STEPS studies (S1 Table). Iran started the NCD risk factor surveillance project in 2005. Since then, seven large-scale cross-sectional multicenter surveys have been conducted using the recommended WHO STEPS tool [13,14]. As WHO suggested, countries can modify risk factors and other variables of the study to reach their local and regional interests [15]. The STEPS studies consist of three different levels of risk factor assessment, including collecting information by questionnaire (step 1), physical measurements (step 2), and biochemical measurements (step 3). Four out of seven STEPS studies conducted in Iran performed all three steps (2005, 2007, 2011, and 2016). While the rest of the three studies, conducted in 2006, 2008, and 2009, only covered the first and second steps of the risk assessment.

Each STEPS study was done with a close relationship between the central team appointed by the Iranian Ministry of Health and Medical Education and its affiliated medical universities. Unfortunately, in 2016 one province (Qom) refused to participate in the survey. Medical university officers selected members of the interview teams and district supervisors by setting specific criteria, including strong communication skills and familiarity with geographical area and their culture. Comprehensive training workshops were held at national and subnational levels before each study to train medical universities’ officers, supervisors, interviewers, data collectors and other study partners.

Although there are some variations between these seven studies, such as sampling sizes, target age group included in each study, demographic questions used for step 1, and biochemical variables that were assessed, the main features of them are similar and overall consistency is the main strength of STEPS studies. In all of the seven STEPS studies run in Iran, a multistage cluster random sampling, with a probability proportional to the size, was used to obtain a nationally representative sample of the population. Iranian national zip code databank was used as a frame for the selection of the sampling units.

WHO suggested surveillance studies should recruit at least people between 25 and 64 years old, while each country may also include other desirable age groups. Additional youth age group of 15–24 years was included in 2005, 2006, 2007, 2008, 2009 and 2011 STEPS studies while additional age groups of 18-24 and over 65 years were also included in 2016 STEPS study. Blood samples were exclusively taken in those who were >=25 years.

In the first step, participants were asked about their socio-demographic characteristics, behavioral risk factors, medical histories, and history of known risk factors for NCD. The questionnaires were mainly adopted from the one that WHO provided for STEPS studies [15]. The WHO’s questionnaire was translated into Farsi and then translated back into English by independent translators and then its validity and reliability were assessed. Some questions were added or altered to address local features and interests. As we previously mentioned, there are some differences in questions and coding process between seven rounds of STEPS studies. Hence, to merge their data, we adopted the same coding system.

In the second step of all seven studies, all selected participants were invited for physical measurements. All measurements were conducted based on WHO protocols [15]. All instruments and tools were prepared by central committees from the same brands and were standardized and calibrated before the examination. Height was evaluated using a standard height ruler and weight was measured in an upright position by a calibrated scale, while participants had light clothing and had taken their shoes off. Blood pressure was measured in a sitting position after 5 minutes of rest. This assessment was repeated one more time in 2005 and 2006 and two more times in 2007, 2008, 2009, 2011, 2016, each after 5 minutes of rest. In the first two STEPS studies, the average of first and second measurement and in the remaining five studies, an average of second and third assessments were used for interpretation. Quality control of measurements was monitored through periodic assessments conducted by supervisors in each province.

In the third step of STEPS studies conducted in 2005, 2007, 2011, and 2016, all participants, who were >=25 years, were invited for biological sample collection. Samples of venous blood were collected following 12 hours of overnight fasting. All samples were analyzed by reference laboratories with the same method and testing kits. In all four studies, a comprehensive protocol was prepared to ensure the continuance of optimal conditions during sample transfer. Fasting plasma glucose (FPG) and total cholesterol levels were measured in all four mentioned studies. However, high-density lipoprotein cholesterol (HDL) level was only assessed in 2007, 2011, and 2016 (not measured in 2005).

### CVD risk scoring models

In the current study, we assessed both 10-year and 30-year CVD event risk among the Iranian population. To calculate 10-year CVD risk, we used risk prediction models developed by the Framingham study [16], Globorisk study [17,18], and WHO [19]. Globorisk model was developed by researchers from 11 countries, including the current research team, to recalibrate CVD risk scores in different settings. 30-year CVD event risk was calculated based on the Framingham model designed for 30-year risk estimation [20]. All of these models have two subtypes, one based on non-laboratory-predictors (office-based) and the other requiring laboratory parameter results (laboratory-based). Additionally, all of them predict both fatal and nonfatal CVD event risk. However, they have some general differences. Each of these models defines CVD distinctively and incorporates different risk factors for risk prediction. S2 Table summarizes fatal and nonfatal events predicted by each model, the target age groups, and predictors required for risk estimation in each one.

Framingham 10-year risk scores, both non-lab-based and lab-based models, have been previously validated among the Iranian population [21,22]. However, the Framingham 30-year CVD risk scoring model has not been validated among Iranian. The Globorisk study has developed 10-year risk scores for 182 countries, including Iran. Recently, WHO revised its 10-year CVD risk score based on 21 Global Burden of Disease (GBD) regions [19]. It is worth mentioning, both Globorisk and WHO risk scoring models used the Tehran Lipids and Glucose Study (TLGS) [21], a cohort study conducted in Iran, for external validation of their models.

### Variables

To run this study, we gathered required data from previous STEPS studies, including study year, age, sex, geographical location information, smoking status, previous diabetes mellitus (DM) diagnosis, taking anti-hyperglycemic medication (both insulin and oral agents), hypertension treatment, height, weight, mean systolic blood pressure, Fasting plasma glucose (FPG), total cholesterol, High Density Lipoprotein (HDL) cholesterol. Definition of DM was established based on individual self-reports (previous diagnosis of DM by a physician or being under anti-hyperglycemic treatments) or FPG levels of >=126 mg/dl. Body mass index (BMI) was calculated by dividing weight in kilograms by the square of height in meters. As this study was population-based, we did not select participants based on having a previous disease and did not exclude those with prior history of CVD.

### Data preparation

Before beginning statistical analysis, we had to resolve some shortcomings in our dataset.

At subnational level in Iran, the province is the first administrative level and the district is the second. During past decades, the number of provinces in Iran has been changed multiple times, as adjacent districts became independent from one province and founded a new province, or rearranged and merged with an existing province. The administrative changes in provinces produced spatially-misaligned data. Hence, we set the latest administrative division, 31 provinces since 2010, as the reference point and rearranged the STEPS dataset before then by restructuring provinces using district data.

Secondly, human error in data entering was inevitable despite all efforts, which caused us to see some implausible data within our pooled dataset. Hence, we had to identify outliers by setting plausible ranges for some variables, including age, FPG, total cholesterol, HDL, and systolic blood pressure. We defined ranges by using the Third National Health and Nutrition Examination Survey (NHANES III) plausible values [23,24]. We converted the implausible values to missing values at this step.

Lastly, we had to deal with missing values. We aimed to impute data that were missing at random. We used a multiple imputation approach using the Amelia II package in R software [25] for missing values of age, sex, smoking status, hypertension treatment, awareness of DM, systolic blood pressure, anthropometric measurements, FPG, total cholesterol, and HDL. We limited imputation for those who were >=25 years. We did not impute HDL levels for 2005 and all laboratory parameters for 2006, 2008, and 2009, as they were not missing at random.

### Statistical analysis

Framingham’s 10-year CVD event risk was calculated for individuals aged 30 to 74, which helped us have a more robust model for trend analysis. However, we only represented our results for the population aged 40 to 74 years, as WHO and Globorisk models are only applicable to those aged >=40. Additionally, we calculated Framingham’s 30-year CVD risk for individuals aged 25 to 59 years.

Due to the shortcoming of the revised WHO model, which may not be applicable for comparison with historically collected data, we utilized the WHO incident CVD risk score only for data from STEPS 2016 and did not include it in the trend analysis. Office-based risk estimation was done for participants of all seven rounds of STEPS. Yet, laboratory-based CVD risk calculation was limited to participants from 2007, 2011, and 2016 for the Framingham models (no HDL measurement in 2005) and individuals from 2005, 2007, 2011, and 2016 for the Globorisk model.

For STEPS 2016, we calculated the mean 10- and 30-year Framingham risk at national and sub-national levels by sex, area level (rural/urban), and age. We also estimated each risk category’s prevalence in 2016.

We fitted a Random Intercept Mixed Effects model [26] as the primary model in the second step. We used average years of schooling, wealth index, and urbanization rate as the independent variables in all six models. Different Random Intercept Mixed Effects, Random Slope Mixed Effects, and Multilevel Mixed Effects were added and tested to achieve the best model in terms of performance. To reach this goal, we applied the Akaike information criterion (AIC) [27] and Bayesian information criterion (BIC) [28] as the main criteria to compare models. The AIC and BIC values are reported in S3 Table. In all six models, the following structure with three random intercepts for provinces, age groups, and years had the lowest AIC and BIC and was selected as the best one:

$$y_{ijkl}=\beta_{0}+\beta_{1}{sex}_{l}+\beta_{2}{yos}_{ijkl}+\beta_{3}{wi}_{ijkl}+\beta_{4}{urb}_{ijkl}+{PROVINCE}_{i}+{AGE}_{j}+{YEAR}_{k}+\varepsilon_{ijkl}$$

i={0,1,…,30},j={1,2,…,7},k={2005,2006,2007,2008,2009,2011,2016},l=0,1

In this equation, y_ijkl_ is the risk of CVD in province i, age group j, year k, sex l; yos_ijkl_ is the average years of schooling in province i, age group j, year k, sex l, wi_ijkl_ is wealth index in province i, age group j, year k, and sex l, urb_ijkl_ is the urbanization in province i, age group j, year k, sex l, PROVINCE_i_, AGE_j_, and YEAR_k_ are the random intercept terms defined for province i, age group j, and year k, respectively. Lastly, ε_ijkl_ is the model’s residual in the province i age group j year k and gender l, indicating the information that model could not justify.

In the next step, we used an Age-spatio-temporal (AST) model with the chosen structure [29]. We found explicit dependencies across space, time, and age groups that are not negligible at this stage. Thus, we considered the necessary weighting structures in our residual terms. For this purpose, we calculated the difference between predicted and observed CVD outcomes as residual. Then we ran local regression in three matrices of age, sex, and geographic area to calculate the corresponding weight for each data point. Details of the weighting components can be found at (https://rdrr.io/cran/AST/man/AST.html).

We implemented the aforementioned model six times for every response variable (10-year Framingham, 30-year Framingham, Globorisk, and WHO in the laboratory- and office-based forms). For age-standardization, we used Iran’s population structure based on the 2016 census. We calculated the Average Annual Percent Change (AAPC) in estimated age-standardized risk between 2000 and 2030 for each CVD risk scoring model and sex [30,31]. The data cleaning process was conducted in Stata (Stata Statistical Software: Release 14. College Station, TX: StataCorp LP). Also, the statistical analyses regarding the mixed effects and AST models are respectively carried out using "lme4" and "AST" packages in R Statistical Software (version 4.2.1).

### Ethical considerations

In the current study, we used previously gathered data from seven rounds of STEPS studies. All STEPS studies had ethical approval. Written informed consent forms were obtained from all of the participants. The final dataset was de-identified for analysis. Researchers only had access to the de-identified dataset for analysis. Additionally, this study received approval from the ethical committee of the National Institute for Medical Research Development (NIMAD) (ID: IR.NIMAD.REC.1398.307).

## Results

### Baseline characteristics

The baseline characteristics of the included participants of all STEPS surveys, aged 25 to 74 years, are presented in Table 1. Overall, the mean ages of the participants of all surveys were around 45 years, with a fairly balanced sex distribution, except for the 2011 survey, which consisted of 59.7% females. Although the prevalence of current smoking decreased from 19.7% in 2005 to 10.8% in 2016, the prevalence of DM and hypertension treatment increased from 8.7% to 12.3% and from 9.3% to 13%, respectively, in this period (Table 1). The share of the urban population in the included surveys ranged from 54.3% in 2009 to 71.0% in 2016.

**Table 1:** Baseline characteristics of the participants of STEPS surveys.

**Variable**	**STEPS survey**						
	**2005** (n=66,560)	**2006** (n=24,299)	**2007** (n=24,366)	**2008** (n=24,300)	**2009** (n=23,691)	**2011** (n=8,532)	**2016** (n=26,179)
**Demographic characteristics**							
**Age** (year)		44.1±11.5	44.6±11.6	44.5±11.6	44.5±11.6	43.9±11.3	46.2±13.8	44.7±13.1
**Sex** (female)	32,943 (49.4%)	12,126 (49.9%)	12,165 (49.9%)	12,185 (50.1%)	11,828 (49.9%)	5,096 (59.7%)	13,742 (52.4%)
**BMI** (kg/m^2^)	26.2±4.8	26.2±4.8	26.3±5.0	26.2±4.9	26.2±4.9	26.8±5.1	26.9±4.8
**Smoking status** (current)	13,166 (19.7%)	4,887 (20.1%)	4,700 (19.2%)	3,822 (15.7%)	3,632 (15.3%)	963 (11.2%)	2,835 (10.8%)
**DM**	5,848 (8.7%)	1,532 (6.3%)	2,408 (9.8%)	1,716 (7.0%)	1,611 (6.8%)	1,204 (14.1%)	3,231 (12.3%)
**Hypertension treatment**	6,191 (9.3%)	2,192 (9.0%)	2,409 (9.8%)	2,518 (10.3%)	2,427 (10.2%)	1,236 (14.4%)	3,413 (13.0%)
**SBP** (mmHg)	124.7±18.1	121.6±17.5	126.7±18.8	125.5±18.2	125.2±17.6	126.9±18.8	125.5±18.1
**Urban area**	43,069 (64.7%)	15,074 (62.0%)	14,392 (59.0%)	15,047 (61.9%)	12,867 (54.3%)	5,983 (70.1%)	18,594 (71.0%)
**Total cholesterol** (mg/dL)	201.5±40.4	-	191.1±43.2	-	-	183.7±37.6	163.4±32.7
**HDL-C** (mg/dL)	-	-	43.1±11.1	-	-	44.1±9.8	41.0±9.9

Continuous variables are shown in means ± standard deviations and categorical variables are presented in number (percentage).

DM: Diabetes Mellitus; HDL-C: High Density Lipoprotein cholesterol; SBP: Systolic Blood Pressure.

We calculated the CVD risk for all eligible age groups using four previously described office-based models; nevertheless, the laboratory-based risk score could not be calculated in 2006, 2008, and 2009 for none of the laboratory-based models and 2005 for only two laboratory-based Framingham scorings. The number of valid CVD risks calculated by the employed risk scoring models in each STEPS survey can be found in the S4 Table.

### Insights from the 2016 STEPS survey

The 2016 STEPS survey is the most recent national survey on NCD risk factors. In 2016, the age-standardized 10-year CVD event risk was 6.0% and 7.1% among females based on laboratory- and office-based Framingham models, respectively. Framingham laboratory- and office-based age-standardized 10-year risk among males was 10.6% and 12.1%, respectively. This survey revealed that among the Iranian population aged 30 to 74 years, 14.0% and 18.0% (8.0% and 11.8% among females; 20.7% and 24.9% among males) are at great risk (≥20%) of developing CVD in the next 10 years according to the laboratory- and office-based Framingham model, respectively (S5 Table).

In 2016, the age-standardized 30-year risk of incident CVD for females was 16.7% and 17.7%, and for males was 23.5% and 25.7%, based on the laboratory- and office-based models, respectively. Based on laboratory- and office-based Framingham risk scores, 12.7% and 14.9% of individuals aged 25 to 59 years had an estimated risk of ≥45% to develop CVD in the coming 30 years, respectively (9.2% and 11% among females; 16.6% and 19.3% among males) (S5 Table).

**Fig 1:**
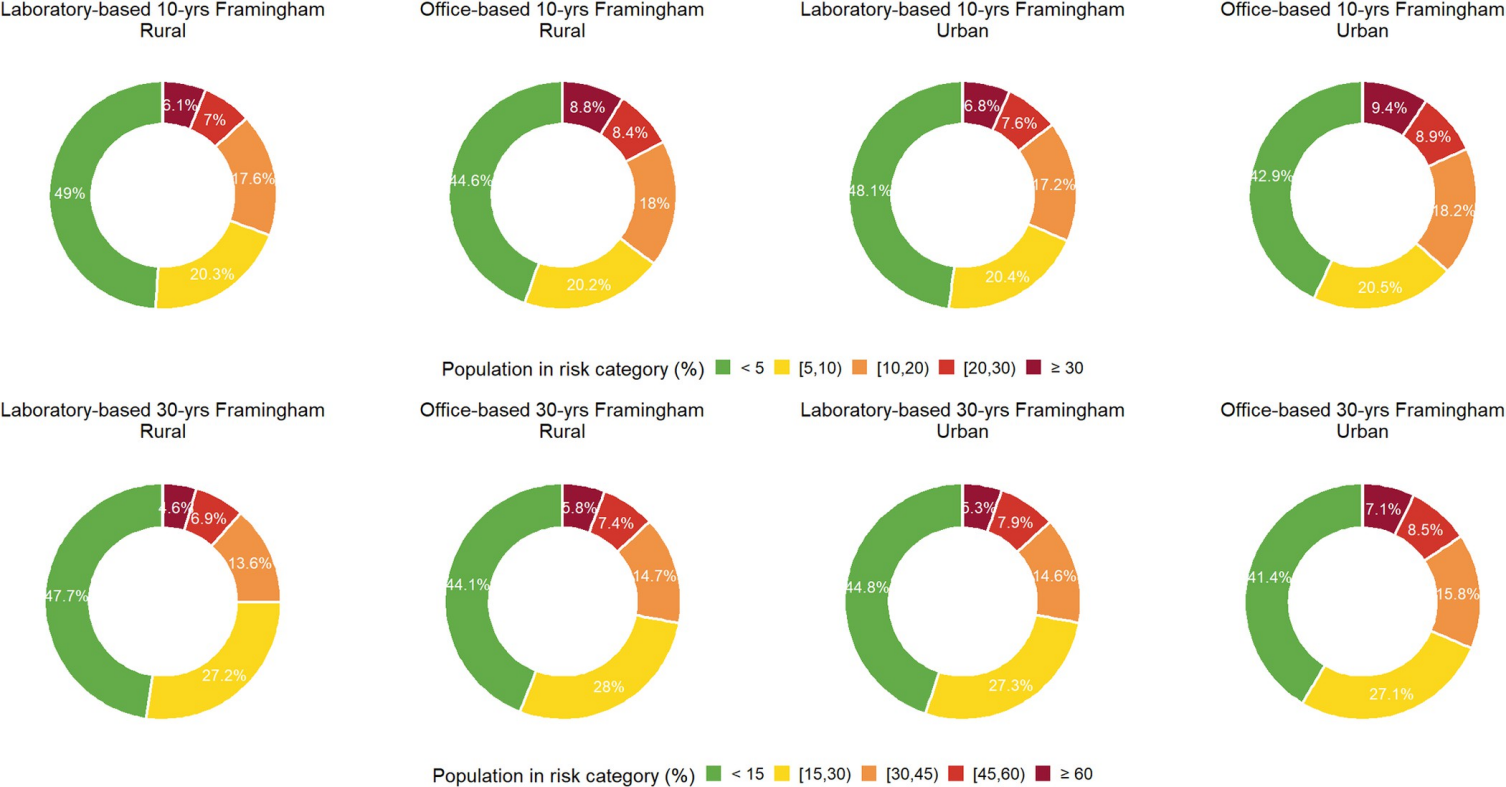
Distribution of 10- and 30-year cardiovascular disease risk based on laboratory- and office-based Framingham models among urban and rural inhabitants in 2016. 10- and 30-year cardiovascular disease risk was calculated for individuals aged 30 to 74 and 25 to 59 years, respectively.

**Fig 2:**
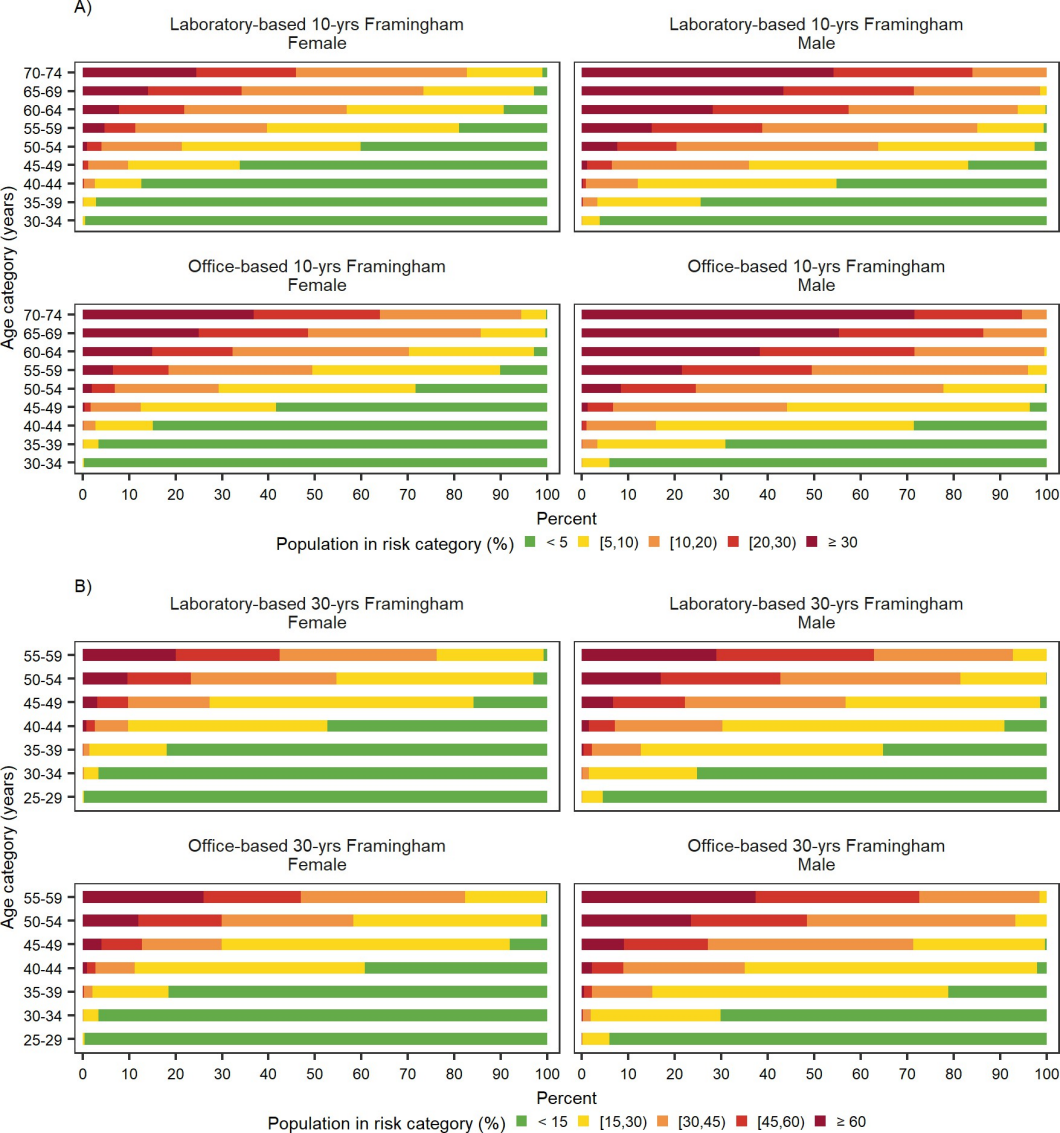
Distribution of 10- and 30- year cardiovascular disease risk based on laboratory- and office-based Framingham models based, by age categories and sex in 2016. A) 10-year CVD risk based on Framingham models, B) 30-year CVD risk based on Framingham models. 10- and 30-year cardiovascular disease risk was calculated for individuals aged 30 to 74 and 25 to 59 years, respectively. CVD: Cardiovascular disease.

Framingham models showed that urban inhabitants are at increased risk of incident CVD compared to rural inhabitants (Fig 1). This rural/urban disparity was more noticeable in the 30-year rather than 10-year Framingham models (Fig 1). See S1 Fig for more detailed findings of CVD event risk in urban and rural areas at subnational level in 2016. Men and older age groups were at greater risk for future CVD based on both 10-year and 30-year Framingham models (Fig 2).

### Trends of CVD risk in Iran

Both Framingham and Globorisk models indicated an increasing trend nationally in age-standardized 10-year risk of incident CVD between 2000 and 2030. We depicted projected age-standardized CVD risk scores in 2000, 2005, 2010, 2015, 2020, 2025, and 2030 in Table 2 and the rest of the projected risk scores in S6 Table.

**Table 2:** Projected age-standardized risks of CVD from 2000 to 2030 and Average Annual Percent Change (AAPC) in estimated age-standardized risk between 2000 and 2030, by each CVD risk scoring model and sex, at national level.

Year	2000	2005	2010	2015	2020	2025	2030	AAPC
**Laboratory-based 10-year Framingham risk score**								
Female	5.9% (2.1-15.1)	6.2% (2.2-15.5)	6.4% (2.2-15.9)	6.5% (2.2-16.0)	7.0% (2.3-16.6)	7.4% (2.4-17.0)	7.8% (2.5-17.4)	0.9%
Male	10.5% (3.6-20.0)	10.8% (3.8-20.3)	11.2% (4.0-20.7)	11.3% (4.0-20.8)	11.8% (4.3-21.3)	12.2% (4.5-21.7)	12.5% (4.6-22.0)	0.6%
**Office-based 10-year Framingham risk score**								
Female	6.1% (2.2-15.5)	6.5% (2.3-16.0)	6.8% (2.4-16.4)	7.1% (2.6-16.8)	7.5% (2.7-17.3)	7.9% (2.9-17.7)	8.2% (3.1-18.0)	1%
Male	11.2% (3.7-20.9)	11.6% (4.0-21.4)	11.8% (4.2-21.6)	12.2% (4.5-21.9)	12.4% (4.7-22.2)	12.8% (5.0-22.5)	13.1% (5.2-22.9)	0.5%
**Laboratory-based 10-year Globorisk risk score**								
Female	10.9% (3.5-22.3)	12.0% (3.7-23.4)	11.3% (3.5-22.8)	11.0% (3.4-22.5)	11.8% (3.6-23.2)	12.0% (3.6-23.5)	12.3% (3.6-23.7)	0.25%
Male	11.6% (3.1-23.1)	12.8% (3.3-24.2)	12.2% (3.2-23.6)	11.9% (3.1-23.4)	12.7% (3.3-24.1)	13.0% (3.4-24.4)	13.3% (3.5-24.7)	0.3%
**Office-based 10-year Globorisk risk score**								
Female	11.3% (3.9-22.7)	12.3% (4.2-23.8)	11.7% (3.9-23.1)	11.7% (3.8-23.2)	12.5% (4.0-24.0)	12.8% (4.1-24.3)	13.0% (4.1-24.5)	0.4%
Male	11.6% (3.3-23.0)	12.8% (3.7-24.2)	12.3% (3.5-23.7)	12.5% (3.5-23.9)	13.4% (3.9-24.9)	13.9% (4.0-25.4)	14.4% (4.3-25.9)	0.6%
**Laboratory-based 30-year Framingham risk score**								
Female	18.0% (7.3-32.1)	18.5% (7.4-32.6)	18.8% (7.4-32.9)	18.7% (7.4-32.9)	19.5% (7.7-33.7)	19.9% (7.8-34.0)	20.3% (7.9-34.4)	0.4%
Male	22.5% (10.3-36.7)	23.3% (10.7-37.4)	23.7% (11.0-37.9)	23.8% (11.0-38.0)	24.7% (11.5-38.8)	25.3% (11.8-39.4)	25.9% (12.3-40.1)	0.4%
**Office-based 30-year Framingham risk score**								
Female	14.3% (5.0-29.0)	16.5% (5.9-31.3)	17.9% (6.5-32.7)	19.4% (7.1-34.2)	21.6% (8.2-36.4)	23.5% (9.1-38.3)	25.3% (10.5-40.1)	2%
Male	22.5% (10.3-37.3)	24.4% (11.5-39.2)	25.3% (12.1-40.1)	26.3% (12.6-41.1)	28.0% (13.8-42.8)	29.4% (14.9-44.2)	30.7% (16.0-45.5)	1%

Data are presented as point estimate (95% confidence interval) for projected risks.

**Fig 3:**
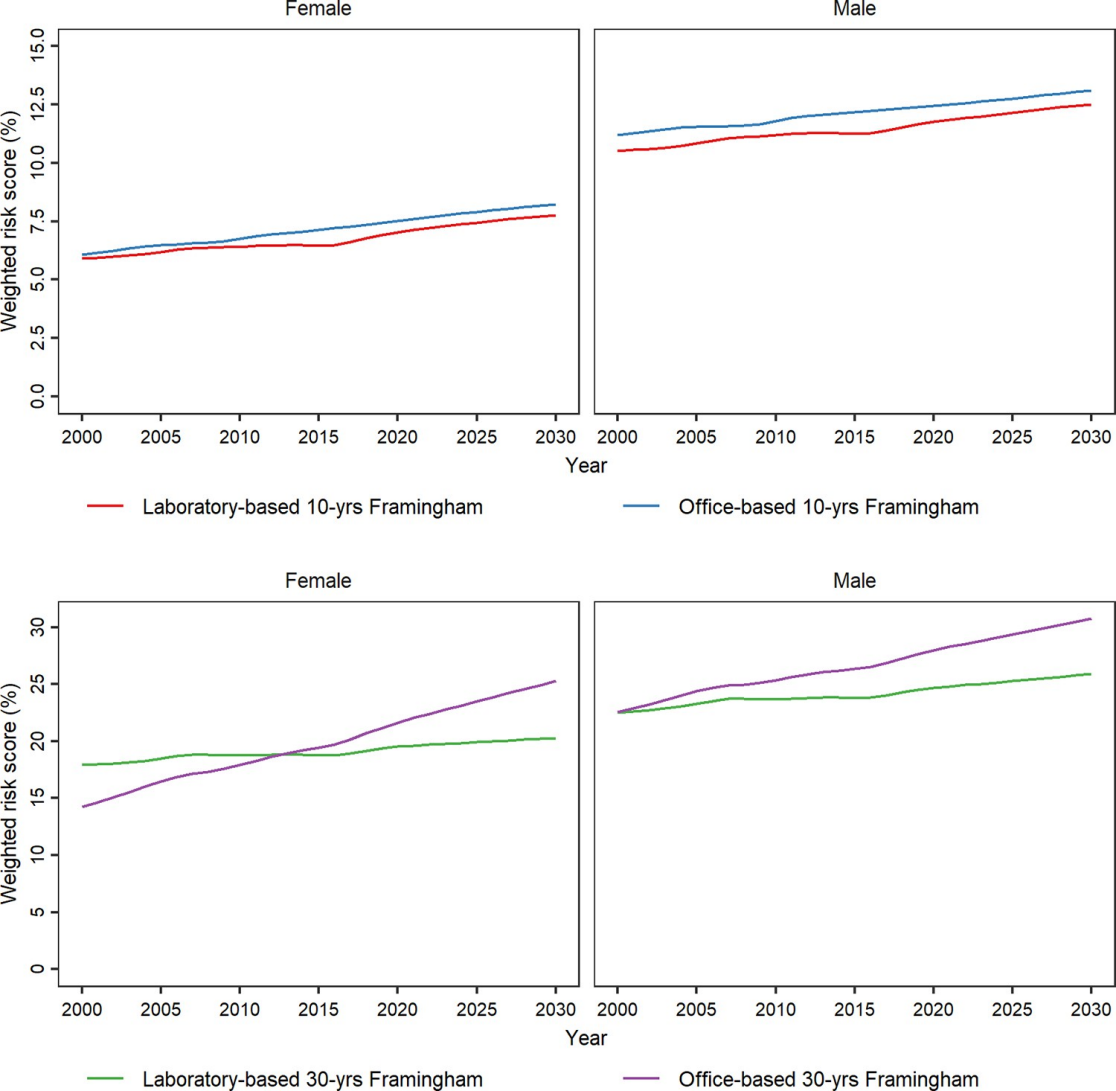
Age-standardized 10- and 30-year cardiovascular disease risk based on laboratory- and office-based Framingham models from 2000 to 2030, by sex. 10- and 30-year cardiovascular disease risk was calculated for individuals aged 30 to 74 and 25 to 59 years, respectively.

The laboratory-based 30-year Framingham risk score revealed that the age-standardized females’ CVD risk will grow from 18.0% (95% CI: 7.3%-32.1%) in 2000 to 20.3% (7.9%-34.4%) in 2030, indicating a 12.8% increase. This model showed that the age-standardized 30-year males’ CVD risk will increase by 15.1% and grow from 22.5% (10.3%-36.7%) in 2000 to 25.9% (12.3%-40.1%) in 2030. The office-based 30-year Framingham model projected that the age-standardized CVD risk will reach 25.3% (10.5%-40.1%) for females and 30.7% (16.0%-45.5%) for males in 2030 (Table 2 and Fig 3).

### Laboratory- versus office-based risk scores

Considering age-standardized CVD risks in females, laboratory-based 10-year models predicted a 0.25% annual increase (Globorisk) to a 0.9% annual increase (Framingham) from 2000 to 2030. Annually, the respective figures for office-based 10-year models were a 0.4% increase (Globorisk) and a 1% increase (Framingham). The predicted AAPC of the age-standardized 10-year CVD risk from 2000 to 2030 ranged from 0.3% (Globorisk) to 0.6% (Framingham) for males with laboratory-based models; nevertheless, the respective figures for office-based models were 0.5% (Framingham) and 0.6% (Globorisk) in males. The predicted age-standardized 10-year CVD risk was consistently higher based on office-based models compared to laboratory-based models between 2000 and 2030 (Table 2 and Fig 3).

Although the projected AAPCs from 2000 to 2030 were not remarkably different between laboratory- and office-based in 10-year models, this difference was eye-catching in the 30-year Framingham model. The laboratory-based model anticipated a 0.25% annual increase in age-standardized CVD risk among females between 2000 to 2030, while the office-based model predicted a 2% annual growth. The respective annual figures for males were 0.4% and 1% (Table 2 and Fig 3).

### Demographic characteristics and CVD risk

**Fig 4:**
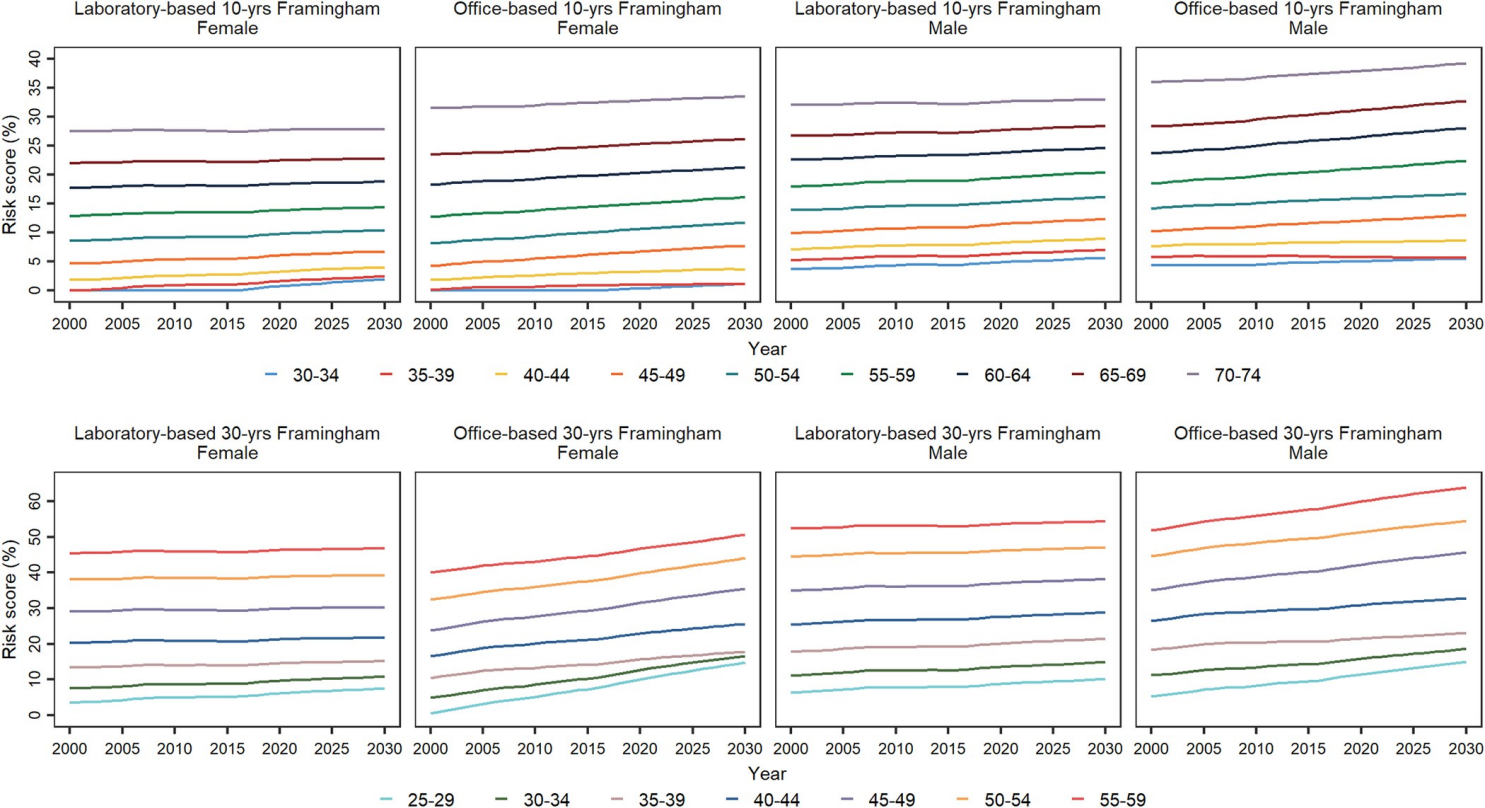
Age-standardized 10- and 30-year cardiovascular disease risk based on laboratory- and office-based Framingham models from 2000 to 2030, by age categories and sex. 10- and 30-year cardiovascular disease risk was calculated for individuals aged 30 to 74 and 25 to 59 years, respectively.

Both 10- and 30-year Framingham models indicated a greater risk of incident CVD in older age groups (Fig 4). Although all Framingham models demonstrated an increasing trend of the CVD risk in all age groups until 2030, 10-year models showed a less pronounced growth in people aged 30-39 years compared to other age categories. Notably, comparing 10- and 30-year Framingham models, the risk of developing CVD events among both females and males ages 30 to 34 and 35 to 39 years is much higher in the next 30 years compared to the next 10 years (Laboratory-based models: 1.9% [95 CI: 0%-11.7%] and 10.8% [0%-24.9%] for 10- and 30-year, respectively, in females aged 30 to 34 years; 5.6% [0%-15.2%] and 15% [0.5%-29.2%] for 10- and 30-year, respectively, in males aged 30 to 34 years) (Fig 4).

### Trends of CVD risk at the subnational level

**Fig 5:**
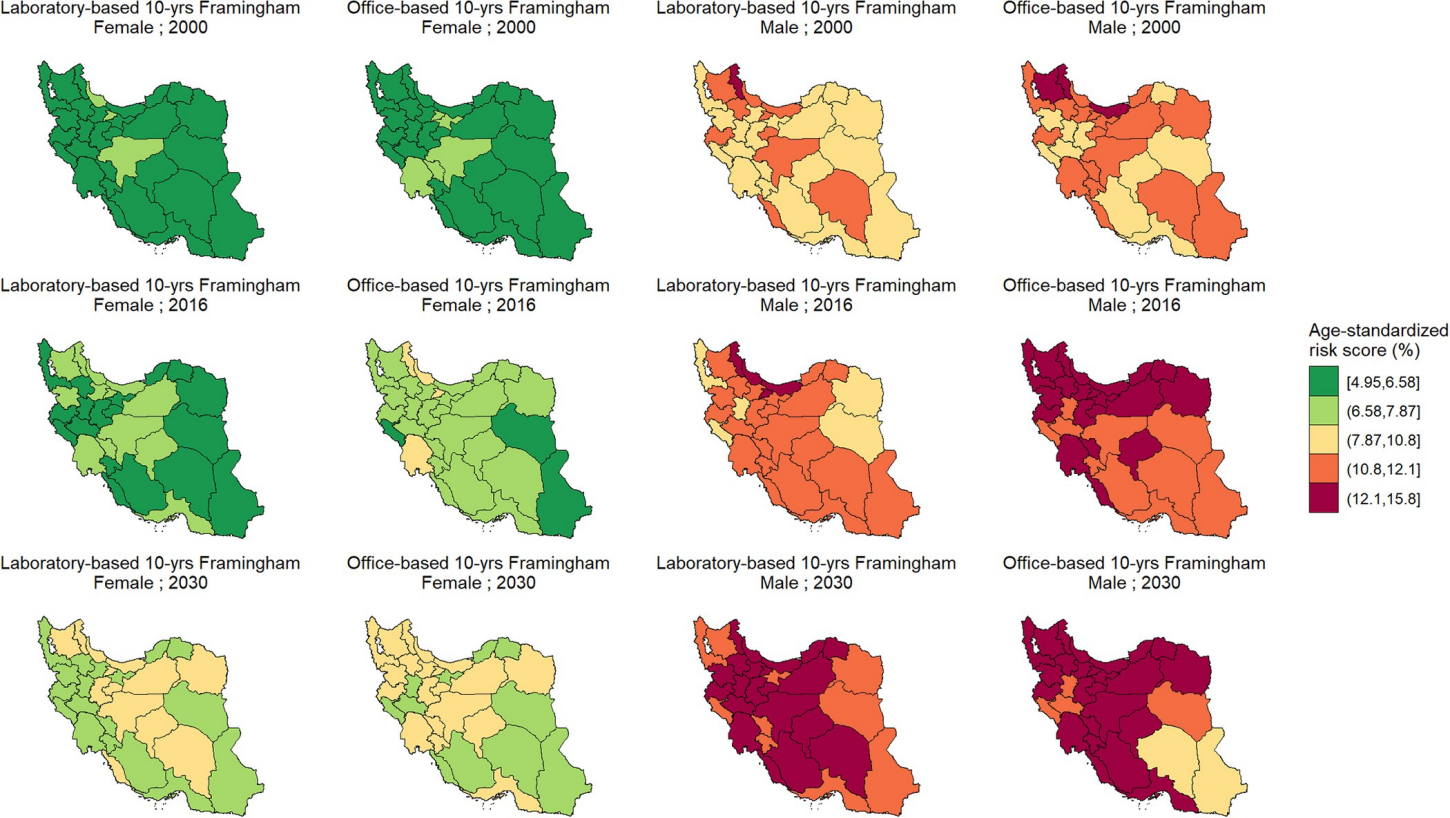
Age-standardized 10-year cardiovascular disease risk based on laboratory- and office-based Framingham models in 2000, 2016, and 2030, by province and sex. 10-year cardiovascular disease risk was calculated for individuals aged 30 to 74. (Contains information from OpenStreetMap and OpenStreetMap Foundation, which is made available under the Open Database License, https://www.openstreetmap.org/copyright).

**Fig 6:**
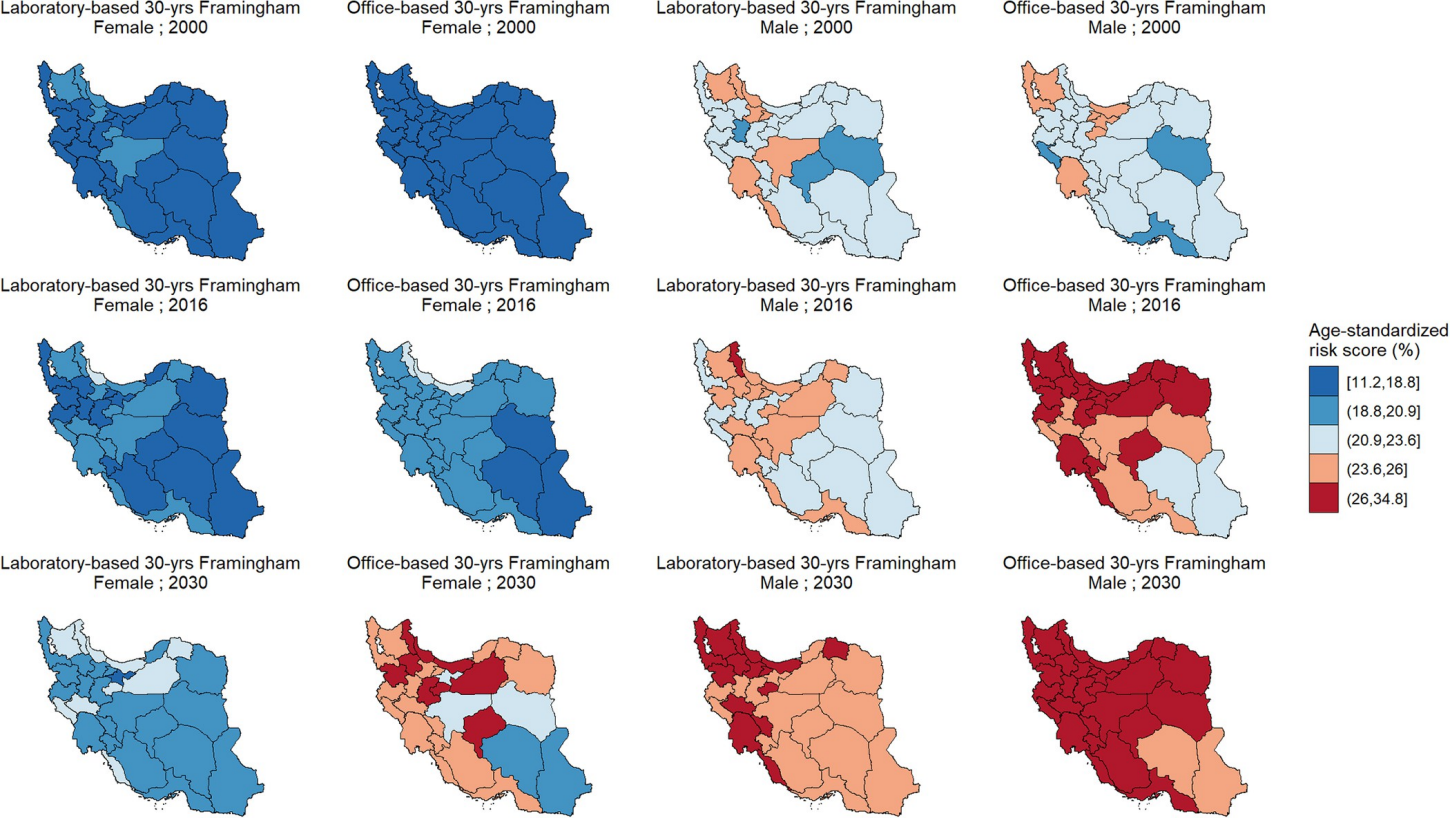
Age-standardized 30-year cardiovascular disease risk based on laboratory- and office-based Framingham models in 2000, 2016, and 2030, by province and sex. 30-year cardiovascular disease risk was calculated for individuals aged 25 to 59. (Contains information from OpenStreetMap and OpenStreetMap Foundation, which is made available under the Open Database License, https://www.openstreetmap.org/copyright).

S7 Table summarizes the lowest and highest projected age-standardized 10- and 30-year CVD event risk in 2000 and 2030 at subnational level, based on Framingham models. According to Framingham models (both laboratory- and office-based), age-standardized 10- and 30- year CVD risk had an increasing trend between 2000 and 2016 and will maintain this upward trend until 2030 (Figs 5 and 6). However, not in all provinces the age-standardized 10- and 30-year incident CVD risk increased at the same pace. For instance, Lorestan, with a 0.2% increase in age-standardized 10-year CVD risk among females, had the lowest percent change between 2000 and 2016, based on Framingham laboratory-based model. In the same setting, Semnan had the highest percent change with a 20.3% increase. Interestingly, South Khorasan had the highest percent change in age-standardized laboratory-based 30-year CVD risk in both females and males (10.4% and 13.1% increase among females and males, respectively), while this province has the lowest projected risk in 2000 in respective groups (S7 Table). Trends of the CVD risk according to Framingham models at the subnational level are presented in the S8 Table.

## Discussion

According to the latest round of STEPS survey in Iran, in 2016, around 15% of the national population, aged 30 to 74, were at ≥20% risk of developing CVD events in the next 10 years. Approximately the same proportion of the population 25 to 59 years was at great (≥45%) risk of incident CVD in the coming 30 years. Based on our trend analysis, we found that the projected 10- and 30-year risk of incident CVD increased in Iran from 2000 to 2016, and it is anticipated that without cardiovascular risk factor modification, this alarming increase will continue, at least, until 2030, according to Framingham and Globorisk models. Our results confirmed that not all specific sub-populations have the same distribution of the CVD risk. As expected, men and older age groups are at a greater risk of incident CVD compared to their counterparts. At the subnational level, eastern provinces had the lowest CVD risk, while northern provinces had the greatest CVD risk from 2000 to 2030. The relative growth of CVD risk in the study period was consistent across laboratory- and office-based models, except for the 30-year Framingham model, in which the predicted increase in CVD risk was profoundly higher in the office-based estimates compared to the laboratory-based.

There are two well-known approaches to prevent CVD, population-wide [32] and high-risk individuals [33] interventions. Geoffrey Rose believes that a minimal alleviation of cardiovascular risk factors at the population level has more robust preventive consequences than a remarkable alleviation of these risk factors among the small number of high-risk individuals [32]. In contrast, individual-based interventions involve people either with a single raised risk factor or at high risk of future CVD for a rigorous risk factor modification. There are long-standing debates on which one of these two strategies should be adopted by health-system policymakers. Critics of approaching high-risk individuals insist that this method will widen inequalities [34], while Lu et al. showed that risk-based intervention, which targeted individuals with a high incident CVD risk score and addressed multiple risk factors at the same time, could considerably reduce CVD rates and racial inequalities in CVD death rates [35]. Additionally, a solely population-based approach requires a robust infrastructure, orchestrated inter-sectoral collaboration, and secure financial support, which are challenging for developing countries like Iran. Hence, in this study, we identified populations at higher risk of future CVD, which can be targeted for rigorous risk factor modification. Furthermore, based on the current study results, most of the population-wide interventions can be established within identified high-risk populations by allocating a greater proportion of our limited resources to those communities [36]. Therefore, understanding the proportion and distribution of high-risk individuals and the trend of national CVD risk may have prime policymaking implications for countries like Iran.

Nationally, the latest STEPS survey in 2016 revealed that nearly 15% of the population, 30 to 74 years and 25 to 59 years, are at great risk of incident CVD events in the next 10 and 30 years, respectively. Thanks to the widespread primary healthcare network in Iran [37], targeting 15% of the adult population who are at the highest risk of future CVD for intense risk factor modification will be a reasonable goal for the Iranian healthcare system. The subnational data provided in this study will aid policymakers in more effective and proportional resource allocation at the subnational level.

In line with previous studies, a greater proportion of populations in urban areas are at risk of developing CVD [38–40]. A popular explanation of the increased risk of CVD among urban inhabitants is that urbanization has an association with lower physical activity and obesity [41]. However, this pattern should be monitored carefully, as sedentary lifestyles and changes in dietary habits are becoming increasingly more common in rural areas as well.

Our results indicate that at any given point between 2000 and 2030, the risk of CVD event, both in 10 years and 30 years, is higher in males than females, which is consistent with other studies [38,42]. Although 10- and 30-year incident CVD risk in both females and males had an upward trend from 2000 to 2030, the growth is more prominent among females based on both 10-year Framingham models and office-based 30-year Framingham model. This trend suggests that cardiovascular risk factor control and modification have been relatively neglected in women compared to men during the past years and underlines the need to raise awareness about CVD in women and close the gap in preventive measures [43].

Our findings contribute to a clearer understanding of the value of office-based risk scoring models. In most individuals with both laboratory- and office-based 10-year risk scores calculated, the office-based value was greater than laboratory-based, and in the remaining individual, the laboratory-based value was slightly greater than office-based. This result highlights that evaluating CVD risk in the primary care setting and the first visit, regardless of the availability of serum lipid levels, has great value in finding individuals with a high chance of CVD events. Hence, utilizing office-based models, CVD risk evaluation should be implemented in the baseline evaluation of individuals without fear of missing high-risk populations and postponing risk calculation to after measuring laboratory values.

Multi-sectoral collaboration is needed for the establishment of the above-mentioned individual-level intervention. Since 2016, a ten-year CVD risk estimation tool (adopted from the previous version of the WHO risk assessment chart) has been embedded in some primary care settings through Iran’s Package of Essential Noncommunicable Diseases (IraPEN) program. However, a new study showed that adherence to regular follow-up in this program was low, reflecting the need to improve the program drastically [44]. Studies showed that early-career physicians with less than five years of experience are less likely to use CVD risk stratification calculators compared to more experienced physicians [45]. Thus, education of primary healthcare providers, especially younger ones, is crucial to the success of this health plan.

One of the main population scale interventions in Iran was the 2014 health transformation plan. After 2014 reform, the Iran’s healthcare expenditure on CVDs increased while out of pocket costs decreased [46]. However, the steady increase in CVD risk in Iran mandates further policies to address this issue. Public health policies to improve physical activity, followed by the interventions addressing salt intake, and tobacco cessation package has been shown as most cost-effective interventions to decrease CVDs in Iran [47].

Further, the current study provides new insights into the importance of 30-year incident CVD risk evaluation in Iran, especially in women and younger individuals [20,48]. Our findings support that, while the 10-year risk of CVD event in these specific individuals may be trivial, calculating 30-year can demonstrate a considerable chance of developing CVD in the same person. Hence, lifelong CVD risk prediction should be adopted in our primary care settings and preventive measures should be started as soon as possible.

As we showed in our subnational analysis, although some provinces, like South Khorasan, had a lower estimated age-standardized 30-year CVD risk in 2000, with the current speed, they will have the highest increase in 2030 among all provinces. It is of high importance for the health system to find the underlying cause of this finding, whether it is due to a relatively higher pace of urbanization there, a higher rate of inequality in healthcare access, lack of plans for cardiovascular risk factor modification, or other possible reason, it should be addressed promptly. Additionally, this result found clear support for the crucial role of trend studies in policymaking, as there was a great possibility that this phenomenon to be missed in a single cross-sectional study.

In this study, we employed a modeling strategy to help Iranian health policymakers to make informed decisions about intense cardiovascular risk factor modification in high-risk individuals. Although our findings may have national rather than international implications, we believe that this study may be a framework to combat CVD and other NCDs in developing countries. Similarly, in another population-level study of CVD risk, Geldsetzer and colleagues delineated the distribution of Indian individuals with the highest CVD risk and cardiovascular risk factors [38]. Similar efforts in other developing countries should be encouraged to address the CVD pandemic. Additionally, regular and consecutive implementation of national surveys like STEPS surveys will provide data on the effectiveness of the previous policies.

### Limitations

In this study, we investigated the trends of the CVD risk in Iran from 2000 to 2030 for the first time that can be adopted in future studies; however, it has several limitations. First, different implementation settings of STEPS surveys in Iran, their non-consecutive implementation, and other inevitable constraints, e.g., refusal of some provinces to participate in the survey, resulted in some drawbacks at the individual level data; nonetheless, we addressed these limitations by employing age-spatio-temporal models and imputation strategies. Second, wide confidence intervals of the estimates resulted in no statistically significant comparison. For the revised WHO CVD risk scoring model, we limited our analysis to only 2016 due to the limitations of this model.

Iran underwent two major healthcare reforms in 2004 (the Universal Rural Health Insurance) and 2014 (the Health Transformation Plan). The 2004 reform was focused on improving children’s health, while the 2014 reform aimed to control noncommunicable diseases, including cardiovascular diseases, by integrating multifactorial approaches to alleviate the atherosclerosis risk factors. The impact of these health policies on CVDs is hard to be separated from the impact of sanctions implemented in 2011 and intensified in 2017. This could impose limitations in translating our predictions to health policy making. Theses political changes may have long term impact on CVD incidence that can be measured using difference-in-differences approaches.

## Conclusion

To the best of our knowledge, this is the first population-level study investigating the trend of the CVD risk in Iranian adults based on the available CVD risk stratification strategies and projected this risk up to 2030. We found that the CVD risk increased from 2000 to 2016 and will continue to rise, at least until 2030, if no effective measure is taken. These findings inform health policymakers about the national CVD risk trend and the proportion and subnational distribution of people at the highest risk of developing future CVD. For the sake of CVD prevention in developing countries like Iran, individual-based interventions are more feasible and affordable compared to population-based measures. Therefore, these data contribute to an informed resource allocation by policymakers for intense risk factor modification in high-risk individuals to avert the increasing trend of CVD risk in Iran. According to the comparable performance of laboratory- and office-based models, CVD risk stratification of individuals should not be delayed due to the lack of serum lipid levels.

## Supporting information

S1 Fig Age-standardized 10- and 30-year cardiovascular disease risk based on laboratory- and office-based Framingham models in 2016 in urban and rural areas, by province. 10- and 30-year cardiovascular disease risk was calculated for individuals aged 30 to 74 and 25 to 59 years, respectively. (Contains information from OpenStreetMap and OpenStreetMap Foundation, which is made available under the Open Database License, https://www.openstreetmap.org/copyright).
(TIF)

S1 Table GATHER checklist. (DOCX)

S2 Table Description of Framingham, Globorisk, and WHO CVD risk score models. (DOCX)

S3 Table Calculated values of the Akaike information criterion (AIC) and Bayesian information criterion (BIC) in each risk scoring model. (DOCX)

S4 Table The number of valid data points in each STEPS survey after missing data imputation. (DOCX)

S5 Table Proportion of population with each specific CVD event risk category, based on 10-year and 30-year Framingham models, both sex combined, at national level. (DOCX)

S6 Table Projected age-standardized risks of CVD from 2000 to 2030 by each CVD risk scoring model and sex, at national level. (DOCX)

S7 Table The lowest and the highest CVD risks at the subnational level in 2000 and 2030, based on 10-year and 30-year Framingham models, by sex. (DOCX)

S8 Table Projected age-standardized risks of CVD from 2000 to 2030 by each CVD risk scoring model and sex, at subnational level. (DOCX)

## References

[1] Aminorroaya A, Yoosefi M, Rezaei N, Shabani M, Mohammadi E, Fattahi N, et al. Global, regional, and national quality of care of ischaemic heart disease from 1990 to 2017: a systematic analysis for the Global Burden of Disease Study 2017. European journal of preventive cardiology. 2022;29(2):371–9. Epub 2021/05/28. doi: 10.1093/eurjpc/zwab066 . 34041535

[2] World Health Organization. Noncommunicable diseases country profiles 2018. 2018.

[3] Global age-sex-specific fertility, mortality, healthy life expectancy (HALE), and population estimates in 204 countries and territories, 1950-2019: a comprehensive demographic analysis for the Global Burden of Disease Study 2019. Lancet (London, England). 2020;396(10258):1160–203. Epub 2020/10/19. doi: 10.1016/S0140-6736(20)30977-6 ; PubMed Central PMCID: PMC7566045. 33069325 PMC7566045

[4] United Nations, Department of Economic and Social Affairs, Population Division (2019). World Population Prospects 2019.

[5] Rahmani A, Sayehmiri K, Asadollahi K, Sarokhani D, Islami F, Sarokhani M. Investigation of the Prevalence of Obesity in Iran: a Systematic Review and Meta-Analysis Study. Acta medica Iranica. 2015;53(10):596–607. Epub 2015/11/30. . 26615371

[6] Jackson R, Lawes CM, Bennett DA, Milne RJ, Rodgers A. Treatment with drugs to lower blood pressure and blood cholesterol based on an individual’s absolute cardiovascular risk. Lancet (London, England). 2005;365(9457):434–41. Epub 2005/02/01. doi: 10.1016/S0140-6736(05)17833-7 . 15680460

[7] Arnett DK, Blumenthal RS, Albert MA, Buroker AB, Goldberger ZD, Hahn EJ, et al. 2019 ACC/AHA Guideline on the Primary Prevention of Cardiovascular Disease: A Report of the American College of Cardiology/American Heart Association Task Force on Clinical Practice Guidelines. Circulation. 2019;140(11):e596–e646. Epub 2019/03/19. doi: 10.1161/CIR.0000000000000678 ; PubMed Central PMCID: PMC7734661. 30879355 PMC7734661

[8] NICE. Cardiovascular disease: risk assessment and reduction, including lipid modification. London: National Institute for Health and Care Excellence 2014.

[9] Hilvo M, Dhar I, Lääperi M, Lysne V, Sulo G, Tell GS, et al. Primary cardiovascular risk prediction by LDL-cholesterol in Caucasian middle-aged and older adults: a joint analysis of three cohorts. European journal of preventive cardiology. 2022;29(3):e128–e37. Epub 2021/06/02. doi: 10.1093/eurjpc/zwab075 . 34060615

[10] Aryan Z, Mahmoudi N, Sheidaei A, Rezaei S, Mahmoudi Z, Gohari K, et al. The prevalence, awareness, and treatment of lipid abnormalities in Iranian adults: Surveillance of risk factors of noncommunicable diseases in Iran 2016. Journal of clinical lipidology. 2018;12(6):1471–81.e4. Epub 2018/09/10. doi: 10.1016/j.jacl.2018.08.001 . 30195823

[11] Djalalinia S, Saeedi Moghaddam S, Sheidaei A, Rezaei N, Naghibi Iravani SS, Modirian M, et al. Patterns of Obesity and Overweight in the Iranian Population: Findings of STEPs 2016. Frontiers in endocrinology. 2020;11:42. Epub 2020/03/17. doi: 10.3389/fendo.2020.00042 ; PubMed Central PMCID: PMC7055062. 32174887 PMC7055062

[12] Roth GA, Mensah GA, Johnson CO, Addolorato G, Ammirati E, Baddour LM, et al. Global Burden of Cardiovascular Diseases and Risk Factors, 1990-2019: Update From the GBD 2019 Study. Journal of the American College of Cardiology. 2020;76(25):2982–3021. Epub 2020/12/15. doi: 10.1016/j.jacc.2020.11.010 ; PubMed Central PMCID: PMC7755038. 33309175 PMC7755038

[13] Djalalinia S, Modirian M, Sheidaei A, Yoosefi M, Zokaiee H, Damirchilu B, et al. Protocol Design for Large-Scale Cross-Sectional Studies of Surveillance of Risk Factors of Non-Communicable Diseases in Iran: STEPs 2016. Archives of Iranian medicine. 2017;20(9):608–16. Epub 2017/10/20. . 29048923

[14] World Health Organization. STEPwise approach to chronic disease risk factor surveillance in Iran [cited 2021 January 20]. Available from: https://www.who.int/ncds/surveillance/steps/iran/en/.

[15] World Health Organization. STEPwise approach to surveillance (STEPS) [cited 2021 January 20,]. Available from: https://www.who.int/ncds/surveillance/steps/en/.

[16] D’Agostino RB Sr., Vasan RS, Pencina MJ, Wolf PA, Cobain M, Massaro JM, et al. General cardiovascular risk profile for use in primary care: the Framingham Heart Study. Circulation. 2008;117(6):743–53. Epub 2008/01/24. doi: 10.1161/CIRCULATIONAHA.107.699579 . 18212285

[17] Ueda P, Woodward M, Lu Y, Hajifathalian K, Al-Wotayan R, Aguilar-Salinas CA, et al. Laboratory-based and office-based risk scores and charts to predict 10-year risk of cardiovascular disease in 182 countries: a pooled analysis of prospective cohorts and health surveys. The lancet Diabetes & endocrinology. 2017;5(3):196–213. Epub 2017/01/28. doi: 10.1016/S2213-8587(17)30015-3 ; PubMed Central PMCID: PMC5354360. 28126460 PMC5354360

[18] Hajifathalian K, Ueda P, Lu Y, Woodward M, Ahmadvand A, Aguilar-Salinas CA, et al. A novel risk score to predict cardiovascular disease risk in national populations (Globorisk): a pooled analysis of prospective cohorts and health examination surveys. The lancet Diabetes & endocrinology. 2015;3(5):339–55. Epub 2015/03/31. doi: 10.1016/S2213-8587(15)00081-9 . 25819778 PMC7615120

[19] World Health Organization cardiovascular disease risk charts: revised models to estimate risk in 21 global regions. The Lancet Global health. 2019;7(10):e1332–e45. Epub 2019/09/07. doi: 10.1016/S2214-109X(19)30318-3 ; PubMed Central PMCID: PMC7025029. 31488387 PMC7025029

[20] Pencina MJ, D’Agostino RB Sr., Larson MG, Massaro JM, Vasan RS. Predicting the 30-year risk of cardiovascular disease: the framingham heart study. Circulation. 2009;119(24):3078–84. Epub 2009/06/10. doi: 10.1161/CIRCULATIONAHA.108.816694 ; PubMed Central PMCID: PMC2748236. 19506114 PMC2748236

[21] Khalili D, Hadaegh F, Soori H, Steyerberg EW, Bozorgmanesh M, Azizi F. Clinical usefulness of the Framingham cardiovascular risk profile beyond its statistical performance: the Tehran Lipid and Glucose Study. American journal of epidemiology. 2012;176(3):177–86. Epub 2012/07/21. doi: 10.1093/aje/kws204 . 22814370

[22] Sepanlou SG, Malekzadeh R, Poustchi H, Sharafkhah M, Ghodsi S, Malekzadeh F, et al. The clinical performance of an office-based risk scoring system for fatal cardiovascular diseases in North-East of Iran. PloS one. 2015;10(5):e0126779. Epub 2015/05/27. doi: 10.1371/journal.pone.0126779 ; PubMed Central PMCID: PMC4444120. 26011607 PMC4444120

[23] Centers for Disease Control and Prevention. Third National Health and Nutrition Examination Survey (NHANES III), 1988-94 [cited 2021 January 20,]. Available from: https://wwwn.cdc.gov/nchs/data/nhanes3/1a/exam-acc.pdf.

[24] Centers for Disease Control and Prevention. Third National Health and Nutrition Examination Survey (NHANES III) 2006 [cited 2021 January 20,]. Available from: https://wwwn.cdc.gov/nchs/data/nhanes3/1a/lab-acc.pdf.

[25] Honaker J, King G, Blackwell M. Amelia II: A program for missing data. Journal of statistical software. 2011;45(7):1–47.

[26] Bates D, Mächler M, Bolker B, Walker S. Fitting linear mixed-effects models using lme4. arXiv preprint arXiv:14065823. 2014.

[27] Sakamoto Y, Ishiguro M, Kitagawa G. Akaike information criterion statistics. Dordrecht, The Netherlands: D Reidel. 1986;81(10.5555):26853.

[28] Gideon S. Estimating the dimension of a model. The annals of statistics. 1978;6(2):461.

[29] Sheidaei A, Gohari K, Kasaeian A, Rezaei N, Mansouri A, Khosravi A, et al. National and Subnational Patterns of Cause of Death in Iran 1990-2015: Applied Methods. Archives of Iranian medicine. 2017;20(1):2–11. Epub 2017/01/24. . 28112524

[30] Clegg LX, Hankey BF, Tiwari R, Feuer EJ, Edwards BK. Estimating average annual per cent change in trend analysis. Statistics in medicine. 2009;28(29):3670–82. Epub 2009/10/27. doi: 10.1002/sim.3733 ; PubMed Central PMCID: PMC2843083. 19856324 PMC2843083

[31] Muggeo VM. Comment on ’Estimating average annual per cent change in trend analysis’ by Clegg LX, Hankey BF, Tiwari R, Feuer EJ, Edwards BK, Statistics in Medicine 2009; 28:3670-3682. Statistics in medicine. 2010;29(18):1958–60; author reply 61. Epub 2010/08/04. doi: 10.1002/sim.3850 . 20680988

[32] Rose GA, Khaw K-T, Marmot M. Rose’s strategy of preventive medicine: the complete original text: Oxford University Press, USA; 2008.

[33] Manuel DG, Lim J, Tanuseputro P, Anderson GM, Alter DA, Laupacis A, et al. Revisiting Rose: strategies for reducing coronary heart disease. BMJ (Clinical research ed). 2006;332(7542):659–62. Epub 2006/03/18. doi: 10.1136/bmj.332.7542.659 ; PubMed Central PMCID: PMC1403258. 16543339 PMC1403258

[34] Capewell S, Graham H. Will cardiovascular disease prevention widen health inequalities? PLoS medicine. 2010;7(8):e1000320. Epub 2010/09/03. doi: 10.1371/journal.pmed.1000320 ; PubMed Central PMCID: PMC2927551 Prevention in Populations. HG has long advocated policies to reduce social inequalities. This paper arises from discussions at NICE, but does not necessarily reflect the views of NICE. 20811492 PMC2927551

[35] Lu Y, Ezzati M, Rimm EB, Hajifathalian K, Ueda P, Danaei G. Sick Populations and Sick Subpopulations: Reducing Disparities in Cardiovascular Disease Between Blacks and Whites in the United States. Circulation. 2016;134(6):472–85. Epub 2016/06/22. doi: 10.1161/CIRCULATIONAHA.115.018102 ; PubMed Central PMCID: PMC5001154. 27324491 PMC5001154

[36] Di Cesare M, Khang YH, Asaria P, Blakely T, Cowan MJ, Farzadfar F, et al. Inequalities in non-communicable diseases and effective responses. Lancet (London, England). 2013;381(9866):585–97. Epub 2013/02/16. doi: 10.1016/S0140-6736(12)61851-0 . 23410608

[37] Danaei G, Farzadfar F, Kelishadi R, Rashidian A, Rouhani OM, Ahmadnia S, et al. Iran in transition. Lancet (London, England). 2019;393(10184):1984–2005. Epub 2019/05/03. doi: 10.1016/S0140-6736(18)33197-0 . 31043324

[38] Geldsetzer P, Manne-Goehler J, Theilmann M, Davies JI, Awasthi A, Danaei G, et al. Geographic and sociodemographic variation of cardiovascular disease risk in India: A cross-sectional study of 797,540 adults. PLoS medicine. 2018;15(6):e1002581. Epub 2018/06/20. doi: 10.1371/journal.pmed.1002581 ; PubMed Central PMCID: PMC6007838. 29920517 PMC6007838

[39] Nuotio J, Vähämurto L, Pahkala K, Magnussen CG, Hutri-Kähönen N, Kähönen M, et al. CVD risk factors and surrogate markers - Urban-rural differences. Scandinavian journal of public health. 2020;48(7):752–61. Epub 2019/08/30. doi: 10.1177/1403494819869816 . 31464561

[40] Dolman RC, Wentzel-Viljoen E, Jerling JC, Feskens EJ, Kruger A, Pieters M. The use of predefined diet quality scores in the context of CVD risk during urbanization in the South African Prospective Urban and Rural Epidemiological (PURE) study. Public health nutrition. 2014;17(8):1706–16. Epub 2013/08/21. doi: 10.1017/S1368980013002206 . 23952977 PMC10282358

[41] Yusuf S, Reddy S, Ounpuu S, Anand S. Global burden of cardiovascular diseases: part I: general considerations, the epidemiologic transition, risk factors, and impact of urbanization. Circulation. 2001;104(22):2746–53. Epub 2001/11/28. doi: 10.1161/hc4601.099487 . 11723030

[42] Peiris D, Ghosh A, Manne-Goehler J, Jaacks LM, Theilmann M, Marcus ME, et al. Cardiovascular disease risk profile and management practices in 45 low-income and middle-income countries: A cross-sectional study of nationally representative individual-level survey data. PLoS medicine. 2021;18(3):e1003485. Epub 2021/03/05. doi: 10.1371/journal.pmed.1003485 ; PubMed Central PMCID: PMC7932723. 33661979 PMC7932723

[43] Mosca L, Barrett-Connor E, Wenger NK. Sex/gender differences in cardiovascular disease prevention: what a difference a decade makes. Circulation. 2011;124(19):2145–54. Epub 2011/11/09. doi: 10.1161/CIRCULATIONAHA.110.968792 ; PubMed Central PMCID: PMC3362050. 22064958 PMC3362050

[44] Hadavand Siri F, Khalili D, Hashemi Nazari SS, Ostovar A, Mahdavi A. Adherence to Iran’s Package of Essential Noncommunicable Diseases (IraPEN) Program for Regular Follow-up to Reduce the Risk of Cardiovascular Disease in Healthcare Centers. Iranian Journal of Endocrinology and Metabolism. 2020;22(2):116–26.

[45] Nour-Eldein H, Abdelsalam SA, Nasr GM, Abdelwahed HA. Global Cardiovascular Risk Assessment by Family Physicians in Suez Canal University-Family Medicine Centers-Egypt. Journal of family medicine and primary care. 2013;2(4):365–70. Epub 2013/10/01. doi: 10.4103/2249-4863.123919 ; PubMed Central PMCID: PMC4649869. 26664843 PMC4649869

[46] Alipour V, Zandian H, Yazdi-Feyzabadi V, Avesta L, Moghadam TZ. Economic burden of cardiovascular diseases before and after Iran’s health transformation plan: evidence from a referral hospital of Iran. Cost effectiveness and resource allocation: C/E. 2021;19(1):1. Epub 2021/01/05. doi: 10.1186/s12962-020-00250-8 ; PubMed Central PMCID: PMC7778796. 33390167 PMC7778796

[47] Yousefi M, Dastan I, Alinezhad F, Ranjbar M, Hamelmann C, Ostovar A, et al. Prevention and control of non-communicable diseases in iran: the case for Investment. BMC public health. 2022;22(1):1248. Epub 2022/06/24. doi: 10.1186/s12889-022-13615-w ; PubMed Central PMCID: PMC9229124. 35739516 PMC9229124

[48] Blumenthal RS, Michos ED, Nasir K. Further improvements in CHD risk prediction for women. Jama. 2007;297(6):641–3. Epub 2007/02/15. doi: 10.1001/jama.297.6.641 . 17299201

