# Supplementary figures and images for: Risk of incident cardiovascular diseases at national and subnational levels in Iran from 2000 to 2016 and projection through 2030: Insights from Iran STEPS surveys
Source: PLoS One. 2023 Aug 23;18(8):e0290006. doi: 10.1371/journal.pone.0290006 (PMC10446220; doi:10.1371/journal.pone.0290006)

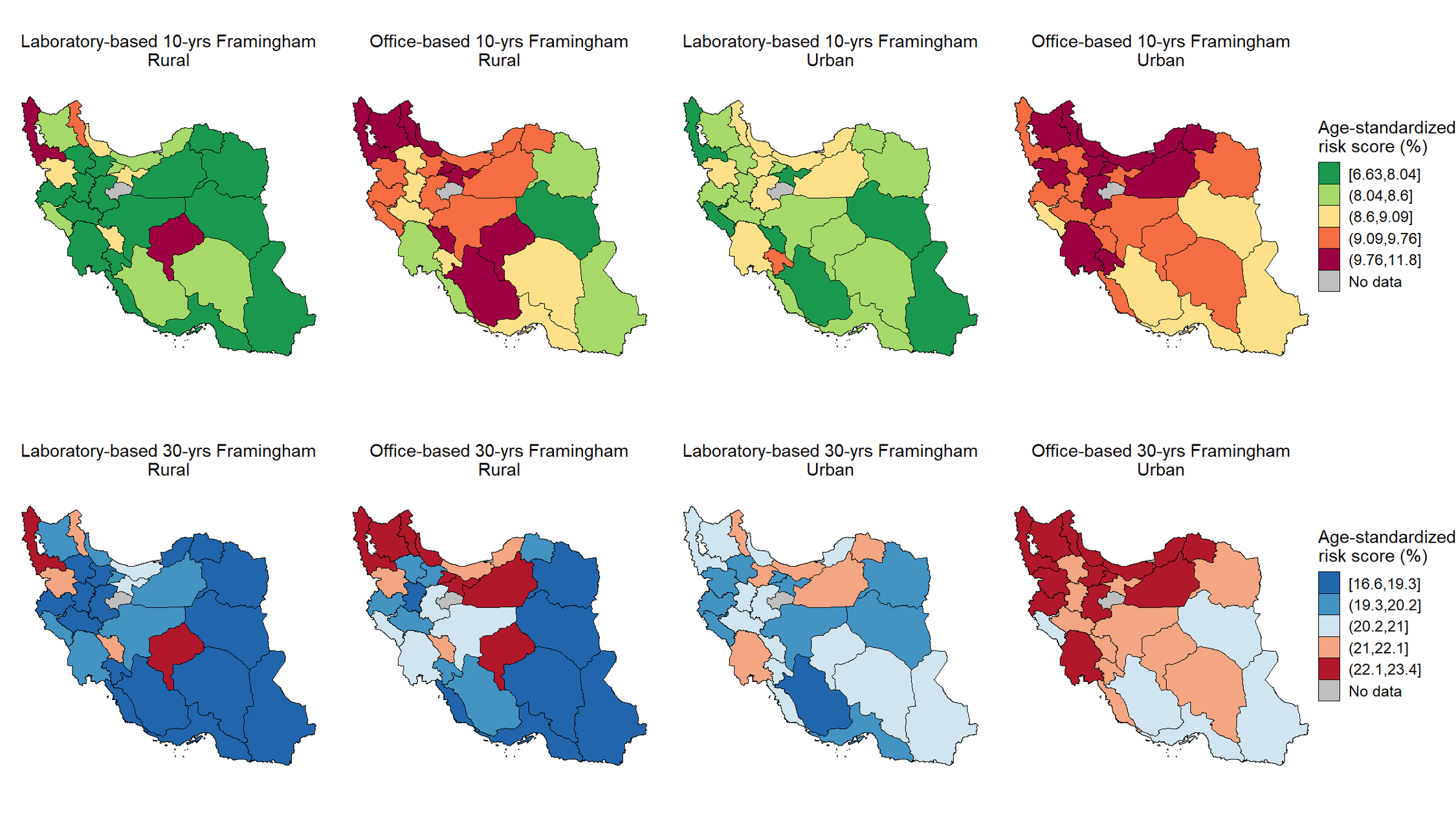

Supplement: S1 Fig — 10- and 30-year cardiovascular disease risk was calculated for individuals aged 30 to 74 and 25 to 59 years, respectively. (Contains information from OpenStreetMap and OpenStreetMap Foundation, which is made available under the Open Database License, https://www.openstreetmap.org/copyright). (TIF) [file pone.0290006.s001.tif]
