# Supplementary material for: Risk of incident cardiovascular diseases at national and subnational levels in Iran from 2000 to 2016 and projection through 2030: Insights from Iran STEPS surveys
Source: PLoS One. 2023 Aug 23;18(8):e0290006. doi: 10.1371/journal.pone.0290006 (PMC10446220; doi:10.1371/journal.pone.0290006)
Supplement: S1 Table — (DOCX) [file pone.0290006.s002.docx]

**S1 Table.** GATHER checklist

| **#** | **GATHER checklist item** | **Reference** |
| --- | --- | --- |
| **1** | Define the indicator(s), populations (including age, sex, and geographic entities), and time period(s) for which estimates were made. | Methods in main text and methods appendix |
| **2** | List the funding sources for the work. | Funding sources |
| **3** | Describe how the data were identified and how the data were accessed. | Methods in main text and methods appendix |
| **4** | Specify the inclusion and exclusion criteria. Identify all ad-hoc exclusions. | Methods in main text and methods appendix |
| **5** | Provide information on all included data sources and their main characteristics. For each data source used, report reference information or contact name/institution, population represented, data collection method, year(s) of data collection, sex and age range, diagnostic criteria or measurement method, and sample size, as relevant. | The STEPS studies are projects launched by the Iranian Ministry of Health and Medical Education of Iran (MOHME) and cannot be posted due to national privacy regulations. Interested and qualified researchers may contact the Ministry of Health and Medical Education of Iran or Non-Communicable Diseases Research Center (Email: [info@ncdrc.info](mailto:info@ncdrc.info) or [Farzadfar3@yahoo.com](mailto:Farzadfar3@yahoo.com)) to access the aggregated datasets of the STEPS studies. |
| **6** | Identify and describe any categories of input data that have potentially important biases (e.g., based on characteristics listed in item 5). | Methods and limitations in main text |
| **7** | Describe and give sources for any other data inputs. | Data of average years of schooling, wealth index, and urbanization rate were used as the independent variables in modeling. Interested and qualified researchers may contact the Non-Communicable Diseases Research Center (Email: [info@ncdrc.info](mailto:info@ncdrc.info) or [Farzadfar3@yahoo.com](mailto:Farzadfar3@yahoo.com)) to access the datasets. |
| **8** | Provide all data inputs in a file format from which data can be efficiently extracted (e.g., a spreadsheet rather than a PDF), including all relevant meta-data listed in item 5. For any data inputs that cannot be shared because of ethical or legal reasons, such as third-party ownership, provide a contact name or the name of the institution that retains the right to the data. | The STEPS studies are projects launched by the Iranian Ministry of Health and Medical Education of Iran (MOHME) and cannot be posted due to national privacy regulations. Interested and qualified researchers may contact the Ministry of Health and Medical Education of Iran or Non-Communicable Diseases Research Center (Email: [info@ncdrc.info](mailto:info@ncdrc.info) or [Farzadfar3@yahoo.com](mailto:Farzadfar3@yahoo.com)) to access the aggregated datasets of the STEPS studies. |
| **9** | Provide a conceptual overview of the data analysis method. A diagram may be helpful. | Methods in main text and methods appendix |
| **10** | Provide a detailed description of all steps of the analysis, including mathematical formulae. This description should cover, as relevant, data cleaning, data pre-processing, data adjustments and weighting of data sources, and mathematical or statistical model(s). | Methods in main text and methods appendix |
| **11** | Describe how candidate models were evaluated and how the final model(s) were selected. | Methods in main text and methods appendix |
| **12** | Provide the results of an evaluation of model performance, if done, as well as the results of any relevant sensitivity analysis. | Methods in main text and methods appendix |
| **13** | Describe methods for calculating uncertainty of the estimates. State which sources of uncertainty were, and were not, accounted for in the uncertainty analysis. | Methods in main text and methods appendix |
| **14** | State how analytic or statistical source code used to generate estimates can be accessed. | Interested and qualified researchers may contact the Non-Communicable Diseases Research Center (Email: [info@ncdrc.info](mailto:info@ncdrc.info) or [Farzadfar3@yahoo.com](mailto:Farzadfar3@yahoo.com)) to access the analytic or statistical source codes. |
| **15** | Provide published estimates in a file format from which data can be efficiently extracted. | Main manuscript and appendix file |
| **16** | Report a quantitative measure of the uncertainty of the estimates (e.g. uncertainty intervals). | Main manuscript and appendix file |
| **17** | Interpret results in light of existing evidence. If updating a previous set of estimates, describe the reasons for changes in estimates. | Main manuscript |
| **18** | Discuss limitations of the estimates. Include a discussion of any modelling assumptions or data limitations that affect interpretation of the estimates. | Main manuscript |
