# Supplementary material for: Risk of incident cardiovascular diseases at national and subnational levels in Iran from 2000 to 2016 and projection through 2030: Insights from Iran STEPS surveys
Source: PLoS One. 2023 Aug 23;18(8):e0290006. doi: 10.1371/journal.pone.0290006 (PMC10446220; doi:10.1371/journal.pone.0290006)
Supplement: S2 Table — (DOCX) [file pone.0290006.s003.docx]

**S2 Table.** Description of Framingham, Globorisk, and WHO CVD risk score models.

|  | **Framingham** | | **Globorisk** | | **WHO** | |
| --- | --- | --- | --- | --- | --- | --- |
| **CVD definitions** | | | | | | |
|  | **10-years risk scoring model**  “General” CVD:  Coronary death  Myocardial infarction  Coronary insufficiency  Angina  Ischemic stroke  Hemorrhagic stroke  Transient ischemic attack  Peripheral artery disease  Heart failure | | IHD  Sudden cardiac death  Stroke | | Myocardial infarction  Stroke | |
|  | **30-year risk scoring model**  “General” CVD or  “Hard” CVD:  Coronary death  Myocardial infarction  Stroke | |  |  |  |  |
| **Target age group** | | | | | | |
|  | **10-years risk scoring model**  30 to 74 years old | | 40 to 74 years old | | 40 to 80 years old | |
|  | **30-year risk scoring model**  20 to 59 years old | |  |  |  |  |
| **Required parameters** | | | | | | |
|  | **Laboratory-based** | **Office-based** | **Laboratory-based** | **Office-based** | **Laboratory-based** | **Office-based** |
| **Age** |  |  |  |  |  |  |
| **Sex** |  |  |  |  |  |  |
| **Systolic blood pressure** |  |  |  |  |  |  |
| **Hypertension treatment** |  |  |  |  |  |  |
| **Smoking** |  |  |  |  |  |  |
| **DM** |  |  |  |  |  |  |
| **Total Cholesterol** |  |  |  |  |  |  |
| **HDL** |  |  |  |  |  |  |
| **Body Mass Index** |  |  |  |  |  |  |
| **Country** |  |  |  |  |  |  |
| **GBD region** |  |  |  |  |  |  |

DM: Diabetes Mellitus; GBD: Global burden of disease; HDL: High-density lipoprotein cholesterol.
