# Supplementary material for: Risk of incident cardiovascular diseases at national and subnational levels in Iran from 2000 to 2016 and projection through 2030: Insights from Iran STEPS surveys
Source: PLoS One. 2023 Aug 23;18(8):e0290006. doi: 10.1371/journal.pone.0290006 (PMC10446220; doi:10.1371/journal.pone.0290006)
Supplement: S3 Table — (DOCX) [file pone.0290006.s004.docx]

**S3 Table.** Calculated values of the Akaike information criterion (AIC) and Bayesian information criterion (BIC) in each risk scoring model

| CVD risk scoring model | AIC | BIC |
| --- | --- | --- |
| Laboratory-based 10-yrs Framingham | 6143.4 | 6189.3 |
| Office-based 10-yrs Framingham | 14051 | 14104.8 |
| Laboratory-based 30-yrs Framingham | 5637.7 | 5682.6 |
| Office-based 30-yrs Framingham | 13667.4 | 13720.8 |
| Laboratory-based 10-yrs Globorisk | 5250.9 | 5296.8 |
| Office-based 10-yrs Globorisk | 8355 | 8406 |
| Laboratory-based 10-yrs WHO | 3970.6 | 4016.4 |
| Office-based 10-yrs WHO | 6671.5 | 6722.5 |
