# Supplementary material for: Risk of incident cardiovascular diseases at national and subnational levels in Iran from 2000 to 2016 and projection through 2030: Insights from Iran STEPS surveys
Source: PLoS One. 2023 Aug 23;18(8):e0290006. doi: 10.1371/journal.pone.0290006 (PMC10446220; doi:10.1371/journal.pone.0290006)
Supplement: S4 Table — (DOCX) [file pone.0290006.s005.docx]

**S4 Table.** The number of valid data points in each STEPS survey after missing data imputation.

| **Risk scoring model** | **STEPS survey** | | | | | | | |
| --- | --- | --- | --- | --- | --- | --- | --- | --- |
|  | **2005** | **2006** | **2007** | **2008** | **2009** | **2011** | **2016** |  |
| **Laboratory-based 10-year Framingham risk score** | - | - | 21,148 | - | - | 7,200 | 22,677 |  |
| **Office-based 10-year Framingham risk score** | 57,566 | 21,073 | 21,148 | 21,092 | 20,447 | 7,200 | 22,677 |  |
| **Laboratory-based 10-year Globorisk risk score** | 41,208 | - | 15,161 | - | - | 5,330 | 15,409 |  |
| **Office-based 10-year Globorisk risk score** | 41,208 | 15,217 | 15,161 | 15,155 | 14,488 | 5,330 | 15,409 |  |
| **Laboratory-based 10-year WHO risk score** | - | - | - | - | - | - | 15,409 |  |
| **Office-based 10-year WHO risk score** | - | - | - | - | - | - | 15,409 |  |
| **Laboratory-based 30-year Framingham risk score** | - | - | 21,368 | - | - | 6,668 | 21,795 |  |
| **Office-based 30-year Framingham risk score** | 58,849 | 21,245 | 21,368 | 21,364 | 21,375 | 6,668 | 21,795 |  |

We used a multiple imputation approach (Amelia II package, R software). Imputation was limited to those who were >=25 years. We also did not impute HDL levels for 2005 and as well as all laboratory parameters for 2006, 2008, and 2009, as they were not missing at random. Thereby, the number of available data points differed among each CVD risk score modeling, as noted in the table.

WHO: World Health Organization
