# Supplementary material for: Risk of incident cardiovascular diseases at national and subnational levels in Iran from 2000 to 2016 and projection through 2030: Insights from Iran STEPS surveys
Source: PLoS One. 2023 Aug 23;18(8):e0290006. doi: 10.1371/journal.pone.0290006 (PMC10446220; doi:10.1371/journal.pone.0290006)
Supplement: S5 Table — (DOCX) [file pone.0290006.s006.docx]

**S5 Table.** Proportion of population with each specific CVD event risk category, based on 10-year and 30-year Framingham models, both sex combined, at national level

| **Risk score category^*^** | **Laboratory-based 10-year Framingham risk score** | **Office-based 10-year Framingham risk score** |
| --- | --- | --- |
| <5% | 48.3% | 43.4% |
| 5-9% | 20.4% | 20.4% |
| 10-19% | 17.3% | 18.2% |
| 20-29% | 7.4% | 8.8% |
| ≥30% | 6.6% | 9.2% |
| **Risk score category^**^** | **Laboratory-based 30-year Framingham risk score** | **Office-based 30-year Framingham risk score** |
| <15% | 45.7% | 42.2% |
| 15-29% | 27.3% | 27.4% |
| 30-44% | 14.3% | 15.5% |
| 45-59% | 7.6% | 8.2% |
| ≥60% | 5.1% | 6.7% |

^*^ Among individuals aged 30 to 74 years

^**^ Among individuals aged 25 to 59 years
