# Supplementary material for: Risk of incident cardiovascular diseases at national and subnational levels in Iran from 2000 to 2016 and projection through 2030: Insights from Iran STEPS surveys
Source: PLoS One. 2023 Aug 23;18(8):e0290006. doi: 10.1371/journal.pone.0290006 (PMC10446220; doi:10.1371/journal.pone.0290006)
Supplement: S6 Table — (DOCX) [file pone.0290006.s007.docx]

**S6 Table.** Projected age-standardized risks of CVD from 2000 to 2030 by each CVD risk scoring model and sex, at national level

| **Year** | **Sex** | **Laboratory-based 10-year Framingham risk score** | **Office-based 10-year Framingham risk score** | **Laboratory-based 30-year Framingham risk score** | **Office-based 30-year Framingham risk score** | **Laboratory-based 10-year Globorisk risk score** | **Office-based 10-year Globorisk risk score** |
| --- | --- | --- | --- | --- | --- | --- | --- |
| 2000 | Female | 5.9% (2.1-15.1) | 6.1% (2.2-15.5) | 18% (7.3-32.1) | 14.3% (5-29) | 10.9% (3.5-22.3) | 11.3% (3.9-22.7) |
|  | Male | 10.5% (3.6-20) | 11.2% (3.7-20.9) | 22.5% (10.3-36.7) | 22.5% (10.3-37.3) | 11.6% (3.1-23.1) | 11.6% (3.3-23) |
| 2001 | Female | 5.9% (2.1-15.2) | 6.2% (2.2-15.6) | 18% (7.3-32.1) | 14.7% (5.2-29.4) | 11% (3.5-22.5) | 11.4% (3.9-22.8) |
|  | Male | 10.6% (3.7-20.1) | 11.3% (3.8-21) | 22.6% (10.3-36.8) | 22.9% (10.5-37.6) | 11.8% (3.1-23.2) | 11.7% (3.3-23.1) |
| 2002 | Female | 6% (2.1-15.2) | 6.2% (2.2-15.7) | 18.1% (7.3-32.2) | 15.1% (5.3-29.8) | 11.2% (3.5-22.6) | 11.6% (3.9-22.9) |
|  | Male | 10.6% (3.7-20.1) | 11.4% (3.8-21.1) | 22.7% (10.4-36.9) | 23.2% (10.8-38) | 11.9% (3.1-23.4) | 11.9% (3.4-23.3) |
| 2003 | Female | 6% (2.1-15.3) | 6.3% (2.3-15.8) | 18.1% (7.3-32.3) | 15.5% (5.5-30.3) | 11.4% (3.6-22.9) | 11.8% (4-23.2) |
|  | Male | 10.7% (3.7-20.2) | 11.4% (3.9-21.2) | 22.9% (10.5-37) | 23.6% (11-38.4) | 12.2% (3.2-23.6) | 12.1% (3.5-23.6) |
| 2004 | Female | 6.1% (2.2-15.4) | 6.4% (2.3-16) | 18.3% (7.4-32.4) | 16% (5.7-30.8) | 11.7% (3.6-23.2) | 12% (4.1-23.5) |
|  | Male | 10.7% (3.8-20.2) | 11.5% (3.9-21.2) | 23% (10.6-37.2) | 24% (11.2-38.8) | 12.5% (3.2-23.9) | 12.5% (3.6-23.9) |
| 2005 | Female | 6.2% (2.2-15.5) | 6.5% (2.3-16) | 18.5% (7.4-32.6) | 16.5% (5.9-31.3) | 12% (3.7-23.4) | 12.3% (4.2-23.8) |
|  | Male | 10.8% (3.8-20.3) | 11.6% (4-21.3) | 23.3% (10.7-37.4) | 24.4% (11.5-39.2) | 12.8% (3.3-24.2) | 12.8% (3.7-24.2) |
| 2006 | Female | 6.3% (2.2-15.6) | 6.5% (2.3-16.1) | 18.7% (7.5-32.8) | 16.9% (6.1-31.6) | 12% (3.7-23.5) | 12.5% (4.2-23.9) |
|  | Male | 11% (3.9-20.5) | 11.5% (4-21.3) | 23.5% (10.9-37.7) | 24.7% (11.7-39.5) | 12.8% (3.3-24.3) | 12.9% (3.7-24.4) |
| 2007 | Female | 6.4% (2.2-15.7) | 6.6% (2.3-16.1) | 18.8% (7.6-33) | 17.1% (6.2-31.9) | 11.9% (3.7-23.4) | 12.4% (4.2-23.9) |
|  | Male | 11.1% (3.9-20.6) | 11.6% (4-21.3) | 23.7% (11-37.9) | 24.9% (11.8-39.7) | 12.7% (3.3-24.2) | 12.9% (3.7-24.4) |
| 2008 | Female | 6.4% (2.2-15.8) | 6.6% (2.3-16.2) | 18.8% (7.5-33) | 17.3% (6.3-32.1) | 11.7% (3.6-23.2) | 12.2% (4.1-23.6) |
|  | Male | 11.1% (3.9-20.6) | 11.6% (4.1-21.3) | 23.7% (11-37.9) | 25% (11.9-39.8) | 12.5% (3.2-24) | 12.7% (3.7-24.2) |
| 2009 | Female | 6.4% (2.2-15.8) | 6.7% (2.3-16.2) | 18.8% (7.5-32.9) | 17.6% (6.4-32.3) | 11.5% (3.6-23) | 11.9% (4-23.3) |
|  | Male | 11.1% (3.9-20.7) | 11.7% (4.1-21.4) | 23.7% (11-37.9) | 25.1% (12-39.9) | 12.3% (3.2-23.8) | 12.5% (3.6-23.9) |
| 2010 | Female | 6.4% (2.2-15.9) | 6.8% (2.4-16.4) | 18.8% (7.4-32.9) | 17.9% (6.5-32.7) | 11.3% (3.5-22.8) | 11.7% (3.9-23.1) |
|  | Male | 11.2% (4-20.7) | 11.8% (4.2-21.6) | 23.7% (11-37.9) | 25.3% (12.1-40.1) | 12.2% (3.2-23.6) | 12.3% (3.5-23.7) |
| 2011 | Female | 6.4% (2.2-15.9) | 6.9% (2.4-16.5) | 18.8% (7.4-32.9) | 18.3% (6.7-33.1) | 11.3% (3.5-22.8) | 11.6% (3.8-23) |
|  | Male | 11.2% (4-20.8) | 11.9% (4.3-21.7) | 23.7% (11-37.9) | 25.6% (12.2-40.4) | 12.1% (3.1-23.6) | 12.3% (3.5-23.7) |
| 2012 | Female | 6.5% (2.2-15.9) | 6.9% (2.5-16.6) | 18.8% (7.4-32.9) | 18.6% (6.8-33.4) | 11.3% (3.5-22.8) | 11.7% (3.8-23.1) |
|  | Male | 11.3% (4-20.8) | 12% (4.3-21.8) | 23.8% (11-37.9) | 25.9% (12.4-40.7) | 12.1% (3.1-23.6) | 12.4% (3.5-23.8) |
| 2013 | Female | 6.5% (2.2-16) | 7% (2.5-16.7) | 18.8% (7.4-33) | 19% (6.9-33.7) | 11.3% (3.5-22.7) | 11.8% (3.9-23.2) |
|  | Male | 11.3% (4-20.8) | 12.1% (4.4-21.8) | 23.8% (11-38) | 26.1% (12.5-40.9) | 12.1% (3.1-23.6) | 12.5% (3.5-23.9) |
| 2014 | Female | 6.5% (2.2-16) | 7.1% (2.5-16.8) | 18.8% (7.4-32.9) | 19.2% (7-34) | 11.2% (3.4-22.6) | 11.8% (3.8-23.2) |
|  | Male | 11.3% (4-20.8) | 12.1% (4.4-21.9) | 23.8% (11-38) | 26.2% (12.6-41) | 12% (3.1-23.5) | 12.5% (3.6-24) |
| 2015 | Female | 6.5% (2.2-16) | 7.1% (2.6-16.8) | 18.7% (7.4-32.9) | 19.4% (7.1-34.2) | 11% (3.4-22.5) | 11.7% (3.8-23.2) |
|  | Male | 11.3% (4-20.8) | 12.2% (4.5-21.9) | 23.8% (11-38) | 26.3% (12.6-41.1) | 11.9% (3.1-23.4) | 12.5% (3.5-23.9) |
| 2016 | Female | 6.5% (2.2-16) | 7.2% (2.6-16.9) | 18.8% (7.4-32.9) | 19.7% (7.2-34.5) | 10.9% (3.4-22.4) | 11.7% (3.8-23.2) |
|  | Male | 11.3% (4-20.8) | 12.2% (4.5-22) | 23.8% (11-38) | 26.5% (12.7-41.3) | 11.8% (3-23.3) | 12.5% (3.5-23.9) |
| 2017 | Female | 6.6% (2.2-16.2) | 7.3% (2.6-17) | 18.9% (7.4-33.1) | 20.2% (7.4-35) | 11.1% (3.4-22.6) | 11.9% (3.8-23.4) |
|  | Male | 11.4% (4.1-20.9) | 12.3% (4.6-22) | 24% (11.1-38.2) | 26.9% (12.9-41.6) | 12% (3.1-23.5) | 12.7% (3.6-24.2) |
| 2018 | Female | 6.8% (2.3-16.3) | 7.4% (2.7-17.1) | 19.2% (7.5-33.3) | 20.7% (7.7-35.5) | 11.4% (3.5-22.9) | 12.2% (3.9-23.6) |
|  | Male | 11.5% (4.1-21) | 12.3% (4.6-22.1) | 24.3% (11.3-38.5) | 27.3% (13.2-42) | 12.3% (3.2-23.8) | 13% (3.7-24.4) |
| 2019 | Female | 6.9% (2.3-16.4) | 7.4% (2.7-17.2) | 19.4% (7.6-33.5) | 21.2% (7.9-36) | 11.7% (3.5-23.1) | 12.4% (4-23.8) |
|  | Male | 11.6% (4.2-21.2) | 12.4% (4.7-22.1) | 24.5% (11.4-38.7) | 27.7% (13.5-42.4) | 12.6% (3.3-24) | 13.2% (3.8-24.7) |
| 2020 | Female | 7% (2.3-16.6) | 7.5% (2.7-17.3) | 19.5% (7.7-33.7) | 21.6% (8.2-36.4) | 11.8% (3.6-23.2) | 12.5% (4-24) |
|  | Male | 11.8% (4.3-21.3) | 12.4% (4.7-22.2) | 24.7% (11.5-38.8) | 28% (13.8-42.8) | 12.7% (3.3-24.1) | 13.4% (3.9-24.9) |
| 2021 | Female | 7.1% (2.4-16.7) | 7.6% (2.8-17.3) | 19.6% (7.7-33.8) | 22% (8.4-36.8) | 11.9% (3.6-23.3) | 12.6% (4.1-24) |
|  | Male | 11.9% (4.3-21.4) | 12.5% (4.8-22.2) | 24.8% (11.6-38.9) | 28.3% (14-43.1) | 12.8% (3.3-24.2) | 13.5% (3.9-25) |
| 2022 | Female | 7.2% (2.4-16.8) | 7.7% (2.8-17.4) | 19.7% (7.8-33.8) | 22.4% (8.6-37.2) | 11.9% (3.6-23.4) | 12.7% (4.1-24.1) |
|  | Male | 11.9% (4.3-21.5) | 12.6% (4.8-22.3) | 24.9% (11.7-39.1) | 28.6% (14.3-43.3) | 12.9% (3.4-24.3) | 13.6% (3.9-25.1) |
| 2023 | Female | 7.3% (2.4-16.8) | 7.8% (2.9-17.5) | 19.8% (7.8-33.9) | 22.8% (8.8-37.5) | 12% (3.6-23.4) | 12.7% (4.1-24.2) |
|  | Male | 12% (4.4-21.5) | 12.6% (4.9-22.4) | 25% (11.7-39.2) | 28.8% (14.5-43.6) | 12.9% (3.4-24.3) | 13.7% (4-25.2) |
| 2024 | Female | 7.4% (2.4-16.9) | 7.8% (2.9-17.6) | 19.9% (7.8-34) | 23.1% (8.9-37.9) | 12% (3.6-23.4) | 12.8% (4.1-24.2) |
|  | Male | 12.1% (4.4-21.6) | 12.7% (4.9-22.5) | 25.2% (11.8-39.3) | 29.1% (14.7-43.9) | 13% (3.4-24.4) | 13.8% (4-25.3) |
| 2025 | Female | 7.4% (2.4-17) | 7.9% (2.9-17.7) | 19.9% (7.8-34) | 23.5% (9.1-38.3) | 12% (3.6-23.5) | 12.8% (4.1-24.3) |
|  | Male | 12.2% (4.5-21.7) | 12.8% (5-22.5) | 25.3% (11.8-39.4) | 29.4% (14.9-44.2) | 13% (3.4-24.4) | 13.9% (4-25.4) |
| 2026 | Female | 7.5% (2.5-17.1) | 8% (2.9-17.7) | 20% (7.8-34.1) | 23.9% (9.4-38.6) | 12.1% (3.6-23.5) | 12.9% (4.1-24.3) |
|  | Male | 12.2% (4.5-21.8) | 12.8% (5-22.6) | 25.4% (11.9-39.5) | 29.6% (15.1-44.4) | 13.1% (3.4-24.5) | 14% (4.1-25.5) |
| 2027 | Female | 7.6% (2.5-17.2) | 8% (3-17.8) | 20.1% (7.9-34.2) | 24.2% (9.7-39) | 12.1% (3.6-23.6) | 12.9% (4.1-24.4) |
|  | Male | 12.3% (4.5-21.9) | 12.9% (5.1-22.7) | 25.5% (12-39.6) | 29.9% (15.3-44.7) | 13.1% (3.4-24.5) | 14.1% (4.1-25.6) |
| 2028 | Female | 7.7% (2.5-17.2) | 8.1% (3-17.9) | 20.1% (7.9-34.2) | 24.6% (9.9-39.4) | 12.2% (3.6-23.6) | 12.9% (4.1-24.4) |
|  | Male | 12.4% (4.6-21.9) | 13% (5.1-22.7) | 25.6% (12.1-39.8) | 30.2% (15.5-45) | 13.2% (3.5-24.6) | 14.2% (4.2-25.7) |
| 2029 | Female | 7.7% (2.5-17.3) | 8.2% (3-17.9) | 20.2% (7.9-34.3) | 24.9% (10.2-39.7) | 12.2% (3.6-23.7) | 13% (4.1-24.4) |
|  | Male | 12.5% (4.6-22) | 13% (5.2-22.8) | 25.8% (12.2-39.9) | 30.5% (15.7-45.2) | 13.2% (3.5-24.6) | 14.3% (4.2-25.8) |
| 2030 | Female | 7.8% (2.5-17.4) | 8.2% (3.1-18) | 20.3% (7.9-34.4) | 25.3% (10.5-40.1) | 12.3% (3.6-23.7) | 13% (4.1-24.5) |
|  | Male | 12.5% (4.6-22) | 13.1% (5.2-22.9) | 25.9% (12.3-40.1) | 30.7% (16-45.5) | 13.3% (3.5-24.7) | 14.4% (4.3-25.9) |
