# Supplementary material for: Risk of incident cardiovascular diseases at national and subnational levels in Iran from 2000 to 2016 and projection through 2030: Insights from Iran STEPS surveys
Source: PLoS One. 2023 Aug 23;18(8):e0290006. doi: 10.1371/journal.pone.0290006 (PMC10446220; doi:10.1371/journal.pone.0290006)
Supplement: S7 Table — (DOCX) [file pone.0290006.s008.docx]

**S7 Table.** The lowest and the highest CVD risks at the subnational level in 2000 and 2030, based on 10-year and 30-year Framingham models, by sex

| **Risk score** | **2000** | | **2030** | |
| --- | --- | --- | --- | --- |
|  | **Lowest CVD risk** | **Highest CVD risk** | **Lowest CVD risk** | **Highest CVD risk** |
| **Laboratory-based 10-year Framingham risk score** | | | | |
| Female | South Khorasan  5.3% (1.8-14.1) | Gilan  6.8% (2.3-16.2) | South Khorasan  6.6% (2.3-16.3) | Mazandaran  9.3% (2.8-18.9) |
| Male | North Khorasan  9.0% (3.4-18.6) | Ardabil  12.3% (4.1-22.0) | Sistan and Baluchistan  11.2% (4.4-20.8) | Ardabil  14.0% (5.8-23.3) |
| **Office-based 10-year Framingham risk score** | | | | |
| Female | South Khorasan  5.0% (1.5-14.0) | Chahar Mahaal and Bakhtiari  7.4% (2.5-17.2) | South Khorasan  6.7% (2.6-16.5) | Gilan  10.7% (4.3-20.5) |
| Male | South Khorasan  9.9% (3.1-19.4) | Ardabil  12.6% (4.1-22.3) | Kerman  9.9% (4.4-19.7) | Gilan  15.8% (6.6-25.6) |
| **Laboratory-based 30-year Framingham risk score** | | | | |
| Female | South Khorasan  15.2% (5.6-29.2) | Gilan  20.5% (8.9-34.6) | Tehran  18.5% (7.0-32.5) | Gilan  22.3% (9.6-36.2) |
| Male | South Khorasan  19.0% (7.8-33.2) | Gilan  25.5% (12.9-39.5) | Sistan and Baluchistan  24.3% (11.2-38.7) | Ardabil  28.3% (14.4-42.5) |
| **Office-based 30-year Framingham risk score** | | | | |
| Female | South Khorasan  11.2% (3.3-25.8) | Tehran  16.7 (6.4-31.7) | Kerman  19.9% (7.5-34.7) | Gilan  30.2% (15.6-45.0) |
| Male | South Khorasan  18.9% (7.6-33.8) | Tehran  24.8% (12.1-39.5) | Kerman  24.0% (11.3-38.6) | Ardabil  34.8% (19.9-49.4) |
