# Supplementary material for: Risk of incident cardiovascular diseases at national and subnational levels in Iran from 2000 to 2016 and projection through 2030: Insights from Iran STEPS surveys
Source: PLoS One. 2023 Aug 23;18(8):e0290006. doi: 10.1371/journal.pone.0290006 (PMC10446220; doi:10.1371/journal.pone.0290006)
Supplement: S8 Table — (DOCX) [file pone.0290006.s009.docx]

**S8 Table.** Projected age-standardized risks of CVD from 2000 to 2030 by each CVD risk scoring model and sex, at subnational level

| **Year** | **Province** | **Sex** | **Laboratory-based 10-year Framingham risk score** | **Office-based 10-year Framingham risk score** | **Laboratory-based 30-year Framingham risk score** | **Office-based 30-year Framingham risk score** |
| --- | --- | --- | --- | --- | --- | --- |
| 2000 | Alborz | Female | 6.8% (2.2-16.1) | 7.4% (2.5-17.1) | 18.4% (7.8-32.6) | 15.7% (6.1-30.6) |
|  |  | Male | 11.5% (4.1-20.9) | 12.1% (4.3-21.7) | 25% (12.2-39.4) | 24.2% (11.4-39.1) |
| 2000 | Ardabil | Female | 5.8% (2-14.7) | 6.1% (2.2-15.4) | 20% (8.7-34.2) | 14.6% (5.6-29.3) |
|  |  | Male | 12.3% (4.1-22) | 12.6% (4.1-22.3) | 24.9% (12.1-39) | 23.2% (11.1-38.2) |
| 2000 | Azerbaijan, East | Female | 6.1% (2.2-15) | 6.4% (2.3-15.7) | 19.7% (8.3-33.7) | 15.9% (5.7-30.8) |
|  |  | Male | 11.5% (3.7-21) | 12.3% (3.9-22.1) | 24.8% (11.8-39.4) | 24.3% (11.9-39.2) |
| 2000 | Azerbaijan, West | Female | 5.8% (2.2-14.6) | 6.6% (2.6-16.1) | 16.9% (6.7-31.2) | 15.4% (5.6-30) |
|  |  | Male | 10.2% (3.3-19.7) | 11.8% (3.9-21.3) | 21.5% (9.5-36) | 23.6% (11.1-38.2) |
| 2000 | Bushehr | Female | 5.8% (2.2-14.9) | 6% (2.2-15.4) | 19.4% (8.3-33.7) | 14.7% (5.1-29.5) |
|  |  | Male | 10.9% (3.7-20.5) | 11.7% (3.8-21.3) | 24.4% (11.7-38.6) | 22.7% (10.3-37.6) |
| 2000 | Chahar Mahaal and Bakhtiari | Female | 6.4% (2.3-15.8) | 7.4% (2.5-17.2) | 17.6% (6.9-31.7) | 14.8% (5.5-29.5) |
|  |  | Male | 10% (3.8-19.6) | 11.5% (4.2-21.1) | 22.4% (10.2-36.4) | 22.8% (10.6-37.5) |
| 2000 | Fars | Female | 5.6% (2-15) | 6.1% (2.2-15.8) | 17.8% (6.8-31.8) | 14.2% (5.1-28.9) |
|  |  | Male | 9.5% (3.3-18.9) | 10.4% (3.8-20.2) | 22.6% (10.2-36.6) | 22.5% (10.4-37.2) |
| 2000 | Gilan | Female | 6.8% (2.3-16.2) | 6.4% (2.1-15.7) | 20.5% (8.9-34.6) | 14.2% (4.8-28.7) |
|  |  | Male | 11.7% (4.4-21.4) | 11.7% (4.1-21.4) | 25.5% (12.9-39.5) | 23.5% (11.5-38.3) |
| 2000 | Golestan | Female | 5.4% (2-14.6) | 5.9% (2.3-15.4) | 16% (6-29.8) | 14.1% (5.1-29) |
|  |  | Male | 10.3% (3.6-20) | 11.3% (4.1-21.1) | 21.6% (9.4-35.6) | 23.1% (10.5-38.1) |
| 2000 | Hamadan | Female | 5.6% (2-14.8) | 5.9% (2.2-15.5) | 16.3% (6.3-30.4) | 13.6% (4.9-28.2) |
|  |  | Male | 9.1% (3.1-18.8) | 10.3% (3.5-20.1) | 19.8% (8.5-34.1) | 21.4% (9.6-36) |
| 2000 | Hormozgan | Female | 6.4% (2.1-15.4) | 5.7% (1.8-14.9) | 18.4% (7.5-32.4) | 12.7% (4.1-27.3) |
|  |  | Male | 10.2% (3.6-19.7) | 10.3% (3.5-20) | 23% (10.6-37) | 20.3% (8.5-35.2) |
| 2000 | Ilam | Female | 5.6% (1.7-13.8) | 5.4% (1.8-14.1) | 18.2% (7.4-32.1) | 12.4% (4.1-27.3) |
|  |  | Male | 10.1% (3.4-19.4) | 10.1% (3.3-19.8) | 21.9% (10.1-36.2) | 20.7% (8.7-35.4) |
| 2000 | Isfahan | Female | 6.8% (2.4-16) | 6.6% (2.3-15.8) | 19.1% (8-33.3) | 15.6% (5.9-30.5) |
|  |  | Male | 11.4% (3.8-20.9) | 11.7% (3.9-21.6) | 23.7% (11-38) | 23.5% (11.1-38.1) |
| 2000 | Kerman | Female | 6% (1.9-14.9) | 6.2% (2-15.4) | 17.4% (6.7-31.6) | 14% (4.7-28.8) |
|  |  | Male | 10.8% (3.9-20.3) | 11.1% (3.8-20.7) | 21.6% (9.5-35.6) | 21.7% (9.7-36.6) |
| 2000 | Kermanshah | Female | 5.8% (2.3-15) | 6% (2.4-15.5) | 17.8% (7.2-32) | 14.4% (5.1-29.3) |
|  |  | Male | 10.8% (3.6-20.4) | 11.6% (3.8-21.1) | 21.7% (9.7-35.8) | 22.7% (10.5-37.1) |
| 2000 | Khorasan, North | Female | 5.8% (2-15.2) | 6.1% (2.2-15.8) | 18.4% (7.7-32.8) | 13.7% (4.8-28.3) |
|  |  | Male | 9% (3.4-18.6) | 10.5% (3.6-20.2) | 22.7% (10.4-36.8) | 21.2% (9.5-35.9) |
| 2000 | Khorasan, Razavi | Female | 6.1% (1.9-15) | 6.5% (2.1-16) | 16.4% (6.4-30.6) | 13.9% (4.7-28.6) |
|  |  | Male | 10.2% (3.6-19.7) | 10.9% (3.6-20.5) | 21.4% (9.6-35.6) | 22.4% (10.2-36.9) |
| 2000 | Khorasan, South | Female | 5.3% (1.8-14.1) | 5% (1.5-14) | 15.2% (5.6-29.2) | 11.2% (3.3-25.8) |
|  |  | Male | 9.4% (3.4-18.7) | 9.9% (3.1-19.4) | 19% (7.8-33.2) | 18.9% (7.6-33.8) |
| 2000 | Khuzestan | Female | 6% (2.1-15.4) | 6.6% (2.5-16.2) | 18.7% (7.7-32.9) | 15.6% (5.9-30.3) |
|  |  | Male | 10.6% (3.7-20) | 11.5% (4.1-21.4) | 23.8% (11.1-38.1) | 23.8% (11.2-38.6) |
| 2000 | Kohgiluyeh and Boyer-Ahmad | Female | 5.6% (2-14.3) | 5.4% (1.8-14.4) | 17.1% (6.7-31.2) | 13% (4.3-27.5) |
|  |  | Male | 10.5% (3.3-20.1) | 11.2% (3.3-21.1) | 21.2% (9.3-35.2) | 21.6% (9.2-36.3) |
| 2000 | Kurdistan | Female | 5.8% (2.4-15) | 5.8% (2.4-15.3) | 17.9% (7.2-32.2) | 13.8% (4.7-28.5) |
|  |  | Male | 10.5% (3.6-19.9) | 10.7% (3.3-20.4) | 21.7% (9.7-35.8) | 21.7% (9.5-36.3) |
| 2000 | Lorestan | Female | 6.6% (2.2-15.6) | 6.4% (2.1-15.4) | 18.5% (7.8-32.5) | 14.1% (5.1-28.9) |
|  |  | Male | 10.6% (3.7-20) | 10.7% (3.7-20.3) | 23% (10.4-37.1) | 21.8% (9.8-36.4) |
| 2000 | Markazi | Female | 6% (2.1-15.4) | 5.7% (1.9-15.4) | 17.7% (7.1-31.9) | 13.3% (4.2-28) |
|  |  | Male | 9.8% (3.6-19.4) | 10.2% (3.4-19.8) | 22% (10-36.3) | 21.5% (9.6-36.3) |
| 2000 | Mazandaran | Female | 6.3% (2.1-15.5) | 6.6% (2.1-16) | 18.7% (7.6-32.6) | 15.3% (5.6-29.9) |
|  |  | Male | 11.4% (3.9-21) | 12.3% (4.3-22.1) | 23.1% (10.6-37.1) | 24.7% (11.9-39.4) |
| 2000 | Qazvin | Female | 5.8% (2.2-15.2) | 5.3% (2-14.7) | 19% (8.1-33) | 14.1% (4.8-28.6) |
|  |  | Male | 10.7% (3.6-20) | 11.2% (3.5-20.9) | 23.8% (11.3-37.7) | 22.9% (10.4-37.6) |
| 2000 | Qom | Female | 6.2% (2.3-15.6) | 6.4% (2.3-15.7) | 18.9% (8.1-32.9) | 15.6% (5.7-30.5) |
|  |  | Male | 11.9% (3.9-21.4) | 11.9% (3.9-21.7) | 23.2% (10.9-37.5) | 24.6% (11.8-39.3) |
| 2000 | Semnan | Female | 5.7% (2-15.1) | 5.7% (1.9-15.2) | 18.2% (7.5-32.5) | 14.1% (4.8-28.8) |
|  |  | Male | 10.1% (3.6-19.4) | 11.2% (3.9-21) | 22.4% (10.2-36.4) | 22.7% (10.8-37.4) |
| 2000 | Sistan and Baluchistan | Female | 5.7% (2.2-14.6) | 5.6% (2-14.6) | 17.9% (7-32.2) | 14.2% (4.4-28.9) |
|  |  | Male | 10.4% (3.4-19.8) | 10.8% (3.2-20.5) | 22.2% (10.2-36.5) | 21.3% (8.9-36.2) |
| 2000 | Tehran | Female | 6.2% (1.9-14.9) | 7.2% (2.5-16.7) | 17.2% (6.9-31.3) | 16.7% (6.4-31.7) |
|  |  | Male | 9.7% (3.3-19.2) | 11.3% (4.1-21.2) | 22.1% (10-36.2) | 24.8% (12.1-39.5) |
| 2000 | Yazd | Female | 5.7% (2-15.2) | 6.3% (2.1-16) | 15.6% (6.1-29.7) | 14.3% (4.9-29.1) |
|  |  | Male | 9.5% (3.4-19) | 10.7% (3.7-20.5) | 19.7% (8.1-34.1) | 23.1% (10.4-37.5) |
| 2000 | Zanjan | Female | 5.9% (2.3-15) | 5.6% (2.3-14.9) | 17.7% (7-31.5) | 13.7% (4.7-28.6) |
|  |  | Male | 11.4% (4-21) | 11.2% (3.5-20.8) | 22.3% (10-36.3) | 21.7% (9.8-36.4) |
| 2005 | Alborz | Female | 7% (2.5-16.5) | 7.5% (2.8-17.2) | 18.8% (7.8-33.1) | 17.6% (6.7-32.6) |
|  |  | Male | 11.7% (4.2-21.2) | 12.2% (4.5-21.9) | 25.6% (12.4-39.8) | 25.6% (12.2-40.5) |
| 2005 | Ardabil | Female | 6.6% (2.4-16.1) | 7.1% (2.5-16.7) | 20.3% (8.8-34.6) | 17.1% (6.4-31.9) |
|  |  | Male | 11.4% (4-20.9) | 12.3% (4.2-22) | 25.5% (12.5-39.9) | 25.7% (12.7-40.6) |
| 2005 | Azerbaijan, East | Female | 6.5% (2.3-16) | 6.8% (2.5-16.6) | 20% (8.2-34.3) | 17.8% (6.7-32.6) |
|  |  | Male | 11% (4-20.4) | 12.3% (4.5-22) | 25.4% (12-39.6) | 26.1% (12.9-41) |
| 2005 | Azerbaijan, West | Female | 6.1% (2.1-15.3) | 7.1% (2.4-16.5) | 17.5% (6.9-31.7) | 17.4% (6.5-32.1) |
|  |  | Male | 10.3% (3.6-19.9) | 12% (4.2-21.6) | 22.4% (9.9-36.6) | 25.4% (12.4-40.1) |
| 2005 | Bushehr | Female | 6.1% (2.1-15.3) | 6.3% (2.2-15.8) | 19.8% (8.2-34) | 16.6% (5.9-31.5) |
|  |  | Male | 11.3% (3.9-20.8) | 11.7% (4.1-21.5) | 24.9% (11.9-39.2) | 24.1% (11.3-38.8) |
| 2005 | Chahar Mahaal and Bakhtiari | Female | 6.5% (2.3-16) | 7.3% (2.7-17.1) | 18.2% (7.3-32.4) | 16.9% (6.1-31.7) |
|  |  | Male | 10.8% (3.8-20.4) | 12.1% (4.2-21.7) | 23.3% (10.8-37.4) | 24.6% (11.5-39.3) |
| 2005 | Fars | Female | 5.8% (2-14.9) | 6.3% (2.3-15.7) | 18.1% (7.2-32.3) | 15.9% (5.7-30.7) |
|  |  | Male | 10.1% (3.6-19.7) | 11% (4-20.7) | 23.2% (10.6-37.3) | 23.7% (11.1-38.5) |
| 2005 | Gilan | Female | 7% (2.4-16.6) | 6.7% (2.3-16.4) | 21% (9.2-35.1) | 16.9% (6.3-31.9) |
|  |  | Male | 12.2% (4.4-21.7) | 12.3% (4.3-22) | 26% (13-40.1) | 25.5% (12.7-40.4) |
| 2005 | Golestan | Female | 5.7% (2-14.8) | 6.3% (2.3-15.7) | 16.6% (6.4-30.6) | 16.2% (6-31.2) |
|  |  | Male | 10.6% (3.7-20.1) | 11.8% (4.1-21.5) | 22.4% (10.2-36.4) | 24.8% (11.9-39.5) |
| 2005 | Hamadan | Female | 6% (2.1-15.2) | 6.5% (2.3-16) | 16.8% (6.6-30.9) | 16% (5.7-30.9) |
|  |  | Male | 9.6% (3.2-19) | 10.7% (3.7-20.5) | 20.8% (8.9-35) | 23.3% (10.8-38.2) |
| 2005 | Hormozgan | Female | 6.6% (2.4-15.9) | 6% (2.1-15.6) | 18.8% (7.6-33) | 15.4% (5.1-30.2) |
|  |  | Male | 10.7% (3.7-20.4) | 11% (3.6-20.7) | 23.5% (10.8-37.6) | 22.9% (10.2-37.5) |
| 2005 | Ilam | Female | 5.4% (1.9-14.5) | 5.5% (2-14.7) | 18.7% (7.6-32.9) | 14.9% (4.8-29.6) |
|  |  | Male | 10.6% (3.6-20.2) | 10.6% (3.5-20.3) | 22.8% (10.3-36.9) | 22.9% (10-37.8) |
| 2005 | Isfahan | Female | 6.9% (2.4-16.4) | 7.1% (2.5-16.8) | 19.5% (8.1-33.6) | 17.4% (6.5-32.3) |
|  |  | Male | 10.7% (3.8-20.2) | 11.7% (4.2-21.4) | 24.3% (11.5-38.4) | 24.8% (11.8-39.5) |
| 2005 | Kerman | Female | 6% (2.1-15.4) | 6.3% (2.3-15.9) | 17.8% (6.7-32) | 16.1% (5.6-30.8) |
|  |  | Male | 11.3% (4-20.8) | 11.4% (4-21.2) | 22.3% (10-36.5) | 23.3% (10.7-38) |
| 2005 | Kermanshah | Female | 6.1% (2.2-15.4) | 6.5% (2.4-16.1) | 18.3% (7.2-32.5) | 16.7% (5.9-31.3) |
|  |  | Male | 11% (3.9-20.6) | 11.9% (4.2-21.5) | 22.6% (10.3-36.7) | 24.8% (11.6-39.4) |
| 2005 | Khorasan, North | Female | 6% (2.2-15.3) | 6.4% (2.2-15.9) | 19% (7.8-33.2) | 16.3% (5.9-31.2) |
|  |  | Male | 10.2% (3.5-19.8) | 11.7% (3.9-21.4) | 23.5% (10.8-37.4) | 24.1% (11-39) |
| 2005 | Khorasan, Razavi | Female | 6% (2.1-15.1) | 6.8% (2.4-16.4) | 17% (6.6-31.1) | 16% (5.7-30.7) |
|  |  | Male | 10.6% (3.8-20) | 11.2% (3.8-21) | 22.2% (10-36.1) | 24% (11.3-38.8) |
| 2005 | Khorasan, South | Female | 5.6% (1.9-14.5) | 5.3% (1.8-14.6) | 16.1% (6-30.1) | 14% (4.3-28.8) |
|  |  | Male | 10.1% (3.5-19.5) | 10.5% (3.2-20.2) | 20.3% (8.4-34.3) | 22% (9.3-36.9) |
| 2005 | Khuzestan | Female | 6.3% (2.2-15.5) | 7.1% (2.5-16.7) | 19.2% (8-33.3) | 17.4% (6.6-32.2) |
|  |  | Male | 11% (3.8-20.6) | 12.1% (4.3-21.8) | 24.5% (11.4-38.6) | 25.4% (12.3-40.2) |
| 2005 | Kohgiluyeh and Boyer-Ahmad | Female | 6.1% (2.2-15.2) | 6.3% (2.2-15.9) | 17.6% (6.7-31.8) | 15.4% (5.4-30.3) |
|  |  | Male | 10.6% (3.6-20.1) | 10.9% (3.6-20.5) | 22.2% (9.8-36.7) | 24.2% (11.3-39.1) |
| 2005 | Kurdistan | Female | 6.1% (2.2-15.3) | 6.4% (2.3-16) | 18.3% (7.4-32.5) | 16.2% (5.8-30.9) |
|  |  | Male | 10.8% (3.7-20.2) | 11.3% (3.8-21.1) | 22.7% (10.2-36.9) | 24.3% (11.2-39) |
| 2005 | Lorestan | Female | 6.4% (2.3-15.8) | 6.4% (2.4-15.8) | 19.2% (7.8-33) | 16.2% (5.9-31) |
|  |  | Male | 10.9% (3.8-20.3) | 11% (3.8-20.7) | 23.8% (11-38) | 23.6% (11-38.4) |
| 2005 | Markazi | Female | 6.2% (2.1-15.5) | 6.3% (2.1-15.8) | 18.2% (7.3-32.2) | 15.9% (5.4-30.6) |
|  |  | Male | 10.9% (3.7-20.3) | 11.5% (3.7-21.3) | 22.9% (10.5-37) | 24% (11.1-38.9) |
| 2005 | Mazandaran | Female | 6.6% (2.3-16) | 6.9% (2.4-16.7) | 19.3% (8-33.5) | 17.7% (6.6-32.4) |
|  |  | Male | 11.7% (4.2-21.2) | 12.5% (4.5-22.3) | 23.9% (11.3-38) | 26.4% (13-41.3) |
| 2005 | Qazvin | Female | 6.1% (2.1-15.5) | 5.9% (2-15.5) | 19.5% (8.1-33.7) | 16.7% (5.9-31.4) |
|  |  | Male | 10.9% (3.8-20.5) | 11.5% (3.8-21.2) | 24.4% (11.7-38.7) | 24.8% (11.7-39.6) |
| 2005 | Qom | Female | 6.4% (2.2-15.7) | 6.6% (2.4-16.1) | 19.3% (8.2-33.5) | 17.2% (6.2-32) |
|  |  | Male | 11.6% (4.2-21.1) | 11.8% (4.2-21.4) | 23.7% (11.3-37.9) | 25.3% (12.2-40.1) |
| 2005 | Semnan | Female | 6% (2-15.3) | 6.3% (2.2-15.7) | 18.8% (7.8-32.8) | 16.6% (6.1-31.3) |
|  |  | Male | 10.7% (3.8-20.2) | 11.7% (4-21.3) | 23.2% (10.8-37.3) | 24.4% (11.7-39) |
| 2005 | Sistan and Baluchistan | Female | 5.9% (2.1-15.1) | 5.8% (2-15.1) | 18.2% (7.1-32.5) | 15.6% (5.3-30.6) |
|  |  | Male | 10.5% (3.7-20.1) | 10.9% (3.5-20.6) | 22.7% (10.5-37) | 22.6% (10-37.5) |
| 2005 | Tehran | Female | 6.2% (2.2-15.5) | 7.2% (2.8-16.7) | 17.6% (7-31.9) | 18% (6.9-32.7) |
|  |  | Male | 9.8% (3.5-19.4) | 11.3% (4.1-21) | 22.7% (10.4-36.8) | 25.7% (12.4-40.4) |
| 2005 | Yazd | Female | 6.1% (2.1-15.3) | 6.5% (2.3-16.1) | 16.3% (6.3-30.5) | 16.7% (5.9-31.3) |
|  |  | Male | 10.4% (3.5-19.9) | 11.6% (4-21.4) | 20.8% (9.1-34.9) | 24.8% (11.7-39.5) |
| 2005 | Zanjan | Female | 6.1% (2.2-15.4) | 6% (2.1-15.4) | 18.1% (7.3-32.2) | 16.1% (5.8-30.9) |
|  |  | Male | 11.6% (4.2-21.1) | 11.6% (3.9-21.3) | 22.9% (10.4-37.1) | 24% (11-38.7) |
| 2010 | Alborz | Female | 7.2% (2.6-16.8) | 7.7% (2.9-17.5) | 19% (7.8-33.2) | 18.3% (7.1-33.2) |
|  |  | Male | 11.8% (4.3-21.4) | 12.3% (4.5-22.1) | 25.9% (12.6-40.2) | 26.2% (12.6-41.1) |
| 2010 | Ardabil | Female | 6.9% (2.5-16.3) | 7.7% (2.7-17.3) | 20.6% (8.7-34.7) | 19.2% (7.3-34) |
|  |  | Male | 11.9% (4.2-21.3) | 12.8% (4.6-22.4) | 26% (12.7-40.2) | 27.2% (13.6-41.9) |
| 2010 | Azerbaijan, East | Female | 6.7% (2.4-16.1) | 7.1% (2.6-16.9) | 20.2% (8.2-34.2) | 19% (7.2-33.9) |
|  |  | Male | 11.4% (4.1-20.9) | 12.6% (4.7-22.4) | 25.6% (12.2-39.8) | 26.8% (13.3-41.7) |
| 2010 | Azerbaijan, West | Female | 6.2% (2.2-15.6) | 7.4% (2.6-16.9) | 17.8% (6.9-32) | 18.6% (7.2-33.3) |
|  |  | Male | 10.7% (3.8-20.2) | 12.3% (4.4-21.9) | 23% (10.4-37.3) | 26.4% (13-41.1) |
| 2010 | Bushehr | Female | 6.3% (2.1-15.6) | 6.4% (2.3-16) | 20% (8.3-34.2) | 17.8% (6.5-32.7) |
|  |  | Male | 11.7% (4.1-21.2) | 12.1% (4.3-21.8) | 25.2% (11.9-39.4) | 25.1% (12.1-40) |
| 2010 | Chahar Mahaal and Bakhtiari | Female | 6.7% (2.3-16.3) | 7.5% (2.7-17.2) | 18.6% (7.3-32.8) | 18.1% (6.4-32.8) |
|  |  | Male | 11% (3.8-20.7) | 12.1% (4.3-21.9) | 23.8% (11.1-38) | 25.1% (11.9-39.9) |
| 2010 | Fars | Female | 6.1% (2-15.3) | 6.4% (2.3-15.9) | 18.3% (7-32.3) | 17.1% (6.1-31.8) |
|  |  | Male | 10.5% (3.7-20) | 11.1% (4.1-20.9) | 23.5% (10.8-37.5) | 24.5% (11.6-39.2) |
| 2010 | Gilan | Female | 7.3% (2.5-16.8) | 7.3% (2.6-17) | 21.2% (9.2-35.3) | 19.3% (7.5-34.1) |
|  |  | Male | 12.4% (4.6-21.9) | 13% (4.8-22.6) | 26.1% (13-40.2) | 27.2% (13.6-42) |
| 2010 | Golestan | Female | 6% (2.1-15.4) | 6.6% (2.4-16.1) | 17.1% (6.6-31.2) | 17.7% (6.6-32.5) |
|  |  | Male | 10.5% (3.7-20.2) | 11.9% (4.4-21.6) | 22.8% (10.5-36.8) | 25.6% (12.4-40.5) |
| 2010 | Hamadan | Female | 5.9% (2.1-15.3) | 6.6% (2.3-16.3) | 17.2% (6.6-31.2) | 17.5% (6.4-32.4) |
|  |  | Male | 10.5% (3.7-20) | 11% (3.9-20.8) | 21.4% (9.3-35.5) | 24.2% (11.4-39) |
| 2010 | Hormozgan | Female | 7% (2.4-16.5) | 6.4% (2.2-16) | 19.2% (7.6-33.3) | 17.2% (5.8-31.8) |
|  |  | Male | 11.1% (3.8-20.6) | 11.3% (3.8-21) | 23.8% (10.9-38) | 23.8% (10.7-38.5) |
| 2010 | Ilam | Female | 5.6% (1.9-14.9) | 5.8% (2-15.2) | 19.1% (7.7-33.2) | 16.9% (5.4-31.7) |
|  |  | Male | 10.7% (3.4-20) | 11% (3.7-20.8) | 23.1% (10.6-37.2) | 24.4% (11.2-39.2) |
| 2010 | Isfahan | Female | 7.1% (2.4-16.6) | 7.2% (2.6-17) | 19.5% (8-33.7) | 18.2% (6.8-32.9) |
|  |  | Male | 10.9% (3.9-20.5) | 11.8% (4.2-21.6) | 24.5% (11.6-38.8) | 25% (11.8-39.8) |
| 2010 | Kerman | Female | 6.3% (2.2-15.6) | 6.4% (2.3-15.9) | 18.2% (6.8-32.4) | 17% (6-31.7) |
|  |  | Male | 11.7% (4.2-21.3) | 11.5% (4.2-21.3) | 22.7% (10.1-37) | 23.6% (11-38.2) |
| 2010 | Kermanshah | Female | 6.4% (2.2-15.8) | 6.8% (2.4-16.4) | 18.4% (7.1-32.5) | 18.1% (6.6-32.7) |
|  |  | Male | 11.5% (4.1-21) | 12.2% (4.4-22) | 23% (10.5-37.1) | 25.9% (12.4-40.5) |
| 2010 | Khorasan, North | Female | 6.3% (2.2-15.7) | 6.8% (2.3-16.2) | 19.5% (8-33.5) | 18% (6.6-32.9) |
|  |  | Male | 10.7% (3.7-20.1) | 12% (4-21.8) | 24% (11.1-38.2) | 25.5% (12.1-40.3) |
| 2010 | Khorasan, Razavi | Female | 6.2% (2.2-15.5) | 6.9% (2.4-16.5) | 17.3% (6.7-31.5) | 17.3% (6.2-31.9) |
|  |  | Male | 10.5% (3.7-19.9) | 11.6% (4.1-21.3) | 22.6% (10.2-36.8) | 24.8% (11.8-39.5) |
| 2010 | Khorasan, South | Female | 5.8% (1.9-15) | 5.6% (1.8-14.8) | 16.5% (6.1-30.8) | 15.8% (5.1-30.6) |
|  |  | Male | 10.6% (3.6-20.1) | 10.8% (3.5-20.5) | 21% (9-35.3) | 23.3% (10.5-38.2) |
| 2010 | Khuzestan | Female | 6.5% (2.3-16) | 7.5% (2.8-17.2) | 19.4% (7.9-33.6) | 18.7% (7.1-33.4) |
|  |  | Male | 11.4% (4.1-20.9) | 12.1% (4.5-21.9) | 24.9% (11.7-39.1) | 26.4% (12.8-41.4) |
| 2010 | Kohgiluyeh and Boyer-Ahmad | Female | 6.3% (2.2-15.8) | 6.9% (2.3-16.5) | 18% (6.8-32.3) | 17.4% (6-32.2) |
|  |  | Male | 11% (3.8-20.6) | 11.3% (3.9-21) | 22.8% (10.1-37.1) | 25.6% (12.1-40.5) |
| 2010 | Kurdistan | Female | 6.3% (2.3-15.7) | 6.9% (2.4-16.5) | 18.7% (7.4-32.8) | 18% (6.6-32.7) |
|  |  | Male | 11.1% (3.9-20.6) | 11.9% (4.1-21.7) | 23.2% (10.6-37.4) | 26.1% (12.4-40.9) |
| 2010 | Lorestan | Female | 6.7% (2.3-16.2) | 6.5% (2.3-16.1) | 19.5% (7.9-33.7) | 17.6% (6.3-32.4) |
|  |  | Male | 11.3% (4-20.8) | 11.1% (4-21) | 24.2% (11.1-38.3) | 24.4% (11.5-39.3) |
| 2010 | Markazi | Female | 6.5% (2.3-15.9) | 6.8% (2.2-16.5) | 18.5% (7.3-32.6) | 17.8% (6.3-32.4) |
|  |  | Male | 11.2% (3.9-20.6) | 11.9% (4-21.7) | 23.4% (10.8-37.5) | 25.2% (11.9-39.8) |
| 2010 | Mazandaran | Female | 7.2% (2.4-16.7) | 7.2% (2.5-17) | 19.8% (8.2-33.8) | 19.2% (7.2-34) |
|  |  | Male | 12.3% (4.5-21.7) | 12.7% (4.6-22.5) | 24.4% (11.4-38.6) | 27% (13.2-41.8) |
| 2010 | Qazvin | Female | 6.3% (2.2-15.8) | 6.3% (2.2-16) | 19.7% (8-33.7) | 18% (6.6-32.8) |
|  |  | Male | 11.2% (3.9-20.7) | 11.7% (4.1-21.5) | 24.8% (11.8-39) | 25.5% (12-40.1) |
| 2010 | Qom | Female | 6.6% (2.3-16) | 6.8% (2.5-16.4) | 19.5% (8.2-33.8) | 18.5% (6.9-33.2) |
|  |  | Male | 11.8% (4.3-21.3) | 11.9% (4.3-21.6) | 24.1% (11.4-38.2) | 26.1% (12.9-40.8) |
| 2010 | Semnan | Female | 6.7% (2.3-16.2) | 6.9% (2.3-16.6) | 19.3% (7.9-33.4) | 18.2% (6.9-32.9) |
|  |  | Male | 11.4% (4.2-20.9) | 11.7% (4.3-21.4) | 23.7% (11-37.7) | 25% (11.9-39.7) |
| 2010 | Sistan and Baluchistan | Female | 6% (2.1-15.3) | 6% (2.1-15.3) | 18.4% (7.2-32.7) | 16.2% (5.6-31) |
|  |  | Male | 10.9% (3.8-20.5) | 10.9% (3.7-20.6) | 23% (10.6-37.2) | 23% (10.4-37.9) |
| 2010 | Tehran | Female | 6.1% (2.2-15.4) | 6.9% (2.6-16.5) | 17.8% (7-31.9) | 18.3% (7-33.3) |
|  |  | Male | 10.5% (3.8-19.9) | 11.3% (4.2-21.1) | 23% (10.5-37.2) | 25.5% (12.3-40.4) |
| 2010 | Yazd | Female | 6.5% (2.2-15.8) | 6.9% (2.4-16.5) | 16.9% (6.5-31.1) | 18.2% (6.6-33) |
|  |  | Male | 10.9% (3.7-20.5) | 11.8% (4.2-21.6) | 21.6% (9.5-35.7) | 25.6% (12.2-40.4) |
| 2010 | Zanjan | Female | 6.2% (2.2-15.6) | 6.3% (2.2-15.7) | 18.5% (7.2-32.4) | 18% (6.4-32.8) |
|  |  | Male | 12% (4.3-21.6) | 12.1% (4.2-21.8) | 23.5% (10.6-37.6) | 25.6% (12.2-40.6) |
| 2015 | Alborz | Female | 7.4% (2.5-17.1) | 8.4% (3-18.2) | 18.8% (7.6-33) | 19.6% (7.6-34.5) |
|  |  | Male | 12% (4.5-21.6) | 12.8% (4.7-22.7) | 25.9% (12.4-40.1) | 27.5% (13.2-42.3) |
| 2015 | Ardabil | Female | 7% (2.4-16.4) | 8.1% (2.7-17.9) | 20.6% (8.7-34.9) | 21.1% (8.1-36.1) |
|  |  | Male | 12.1% (4.3-21.5) | 13.4% (4.9-23.1) | 26.2% (12.7-40.3) | 28.9% (14.7-43.7) |
| 2015 | Azerbaijan, East | Female | 6.7% (2.4-16.2) | 7.4% (2.7-17.1) | 20% (8.1-34.3) | 20.2% (7.8-35.1) |
|  |  | Male | 11.5% (4.2-21) | 12.8% (4.8-22.5) | 25.5% (12.1-39.8) | 27.4% (13.5-42.3) |
| 2015 | Azerbaijan, West | Female | 6.1% (2.1-15.3) | 7.6% (2.8-17.2) | 17.8% (7-32.2) | 19.7% (7.6-34.4) |
|  |  | Male | 10.8% (3.9-20.2) | 12.5% (4.8-22.3) | 23.3% (10.4-37.5) | 27.4% (13.6-42.1) |
| 2015 | Bushehr | Female | 6.3% (2.1-15.8) | 6.7% (2.4-16.4) | 19.9% (8.1-34.1) | 19.2% (7.3-34) |
|  |  | Male | 11.9% (4.4-21.4) | 12.8% (4.8-22.6) | 25.1% (11.9-39.2) | 26.9% (13.2-41.8) |
| 2015 | Chahar Mahaal and Bakhtiari | Female | 6.7% (2.3-16.2) | 7.7% (2.8-17.3) | 18.7% (7.4-32.9) | 19.3% (6.9-34.3) |
|  |  | Male | 10.8% (3.8-20.3) | 12.2% (4.4-22) | 24% (11.3-38.2) | 25.5% (12.5-40.3) |
| 2015 | Fars | Female | 6% (2.1-15.6) | 6.7% (2.5-16.3) | 18.2% (6.9-32.3) | 18.7% (6.7-33.3) |
|  |  | Male | 10.7% (3.8-20.2) | 11.6% (4.4-21.3) | 23.5% (10.7-37.6) | 25.7% (12.1-40.6) |
| 2015 | Gilan | Female | 7.4% (2.5-16.9) | 8.3% (2.9-18) | 21.1% (9-35.3) | 22% (8.8-36.9) |
|  |  | Male | 12.4% (4.7-21.7) | 13.7% (5.2-23.4) | 26% (12.8-40.1) | 29% (14.8-43.8) |
| 2015 | Golestan | Female | 6.1% (2-15.5) | 6.9% (2.5-16.4) | 17.1% (6.6-31.3) | 19.2% (7.2-34) |
|  |  | Male | 10.8% (3.9-20.4) | 12.3% (4.7-22) | 22.9% (10.5-36.9) | 26.4% (12.8-41.3) |
| 2015 | Hamadan | Female | 6% (2-15.4) | 7% (2.4-16.6) | 17.4% (6.7-31.6) | 19.2% (7-34) |
|  |  | Male | 10.6% (3.8-20.2) | 11.4% (4.2-21.1) | 21.8% (9.6-36) | 25.3% (12.1-40.2) |
| 2015 | Hormozgan | Female | 7.1% (2.4-16.6) | 6.9% (2.4-16.5) | 19.1% (7.5-33.3) | 18.8% (6.3-33.6) |
|  |  | Male | 11% (3.8-20.6) | 11.6% (4-21.3) | 23.7% (10.9-38.1) | 24.7% (11.3-39.4) |
| 2015 | Ilam | Female | 5.8% (1.9-15.3) | 6.2% (2.2-15.9) | 19.3% (7.7-33.4) | 18.9% (6.3-33.7) |
|  |  | Male | 10.2% (3.4-19.8) | 11.3% (4.1-21.1) | 23.1% (10.6-37.2) | 25.6% (12-40.4) |
| 2015 | Isfahan | Female | 7% (2.4-16.6) | 7.5% (2.7-17.3) | 19.2% (7.7-33.3) | 19% (6.9-33.7) |
|  |  | Male | 10.9% (3.8-20.4) | 12% (4.4-21.8) | 24.3% (11.5-38.7) | 25.2% (11.9-40) |
| 2015 | Kerman | Female | 6.4% (2.1-15.8) | 6.6% (2.5-15.8) | 18.1% (6.8-32.4) | 17.4% (6.2-32.1) |
|  |  | Male | 11.7% (4.3-21.2) | 11.3% (4.3-21.1) | 22.8% (10.2-36.9) | 23.3% (10.9-37.7) |
| 2015 | Kermanshah | Female | 6.5% (2.2-15.9) | 7.5% (2.7-17.3) | 18.3% (7-32.5) | 19.6% (7.1-34.2) |
|  |  | Male | 11.4% (4-21) | 12.5% (4.7-22.3) | 23.1% (10.5-37.2) | 27.1% (13.1-41.8) |
| 2015 | Khorasan, North | Female | 6.2% (2.1-15.8) | 7.1% (2.5-16.7) | 19.6% (7.9-33.7) | 19.7% (7.1-34.6) |
|  |  | Male | 10.8% (3.7-20.5) | 12.3% (4.3-22) | 24.2% (11.2-38.5) | 27% (12.8-41.8) |
| 2015 | Khorasan, Razavi | Female | 6.3% (2.1-15.8) | 7.3% (2.6-17) | 17.3% (6.6-31.5) | 19% (6.9-33.8) |
|  |  | Male | 10.4% (3.8-20) | 12.2% (4.4-22) | 22.6% (10.2-36.9) | 25.9% (12.5-40.5) |
| 2015 | Khorasan, South | Female | 5.8% (1.9-15.1) | 5.8% (1.9-15.3) | 16.8% (6.1-31) | 17.4% (5.7-32.2) |
|  |  | Male | 10.6% (3.7-20.1) | 10.8% (3.8-20.5) | 21.5% (9.3-35.6) | 23.7% (10.8-38.6) |
| 2015 | Khuzestan | Female | 6.6% (2.3-16.1) | 8% (3-17.7) | 19.3% (7.8-33.6) | 20.5% (7.8-35.1) |
|  |  | Male | 11.7% (4.1-21.1) | 12.8% (4.8-22.5) | 25% (11.8-39.3) | 27.9% (13.8-42.8) |
| 2015 | Kohgiluyeh and Boyer-Ahmad | Female | 6.3% (2.2-15.7) | 7.5% (2.6-17.3) | 18% (6.7-32.2) | 19.4% (6.8-34) |
|  |  | Male | 11.1% (3.8-20.6) | 11.8% (4.3-21.5) | 23.1% (10.4-37.3) | 27% (12.9-41.7) |
| 2015 | Kurdistan | Female | 6.5% (2.3-15.9) | 7.7% (2.8-17.4) | 18.7% (7.3-32.8) | 20% (7.1-34.8) |
|  |  | Male | 11.3% (3.8-20.7) | 12.6% (4.5-22.4) | 23.6% (10.7-37.6) | 28% (13.8-42.9) |
| 2015 | Lorestan | Female | 6.6% (2.2-16.1) | 6.7% (2.5-16.1) | 19.6% (7.9-33.8) | 18.9% (6.6-33.7) |
|  |  | Male | 11.3% (3.9-20.8) | 11.3% (4.1-21) | 24.4% (11.2-38.6) | 25.2% (11.6-39.8) |
| 2015 | Markazi | Female | 6.5% (2.2-16) | 7.4% (2.4-17) | 18.5% (7.3-32.5) | 19.4% (7.1-34.2) |
|  |  | Male | 11.2% (4-20.7) | 12.3% (4.4-22.1) | 23.5% (10.8-37.8) | 26.2% (12.5-40.9) |
| 2015 | Mazandaran | Female | 7.5% (2.5-17.1) | 7.7% (2.7-17.6) | 19.8% (8.1-33.9) | 20.7% (8-35.5) |
|  |  | Male | 12.5% (4.6-21.9) | 13% (5-22.9) | 24.6% (11.5-38.7) | 27.7% (13.7-42.4) |
| 2015 | Qazvin | Female | 6.7% (2.3-16.3) | 7% (2.5-16.7) | 19.5% (7.8-33.6) | 19.7% (7.2-34.3) |
|  |  | Male | 11.7% (4-21.2) | 12.3% (4.5-22.1) | 24.8% (11.8-39) | 26.2% (12.2-40.9) |
| 2015 | Qom | Female | 6.5% (2.2-15.9) | 7.1% (2.5-16.6) | 19.6% (8.2-33.7) | 20.1% (7.2-34.8) |
|  |  | Male | 11.8% (4.3-21.2) | 12.1% (4.5-22) | 24.1% (11.4-38.4) | 27.1% (13.3-41.9) |
| 2015 | Semnan | Female | 6.8% (2.3-16.4) | 7.4% (2.6-17.1) | 19.3% (8-33.6) | 20.1% (7.8-34.9) |
|  |  | Male | 11.5% (4.2-20.9) | 12.1% (4.4-21.9) | 23.7% (11-37.8) | 25.8% (12.3-40.4) |
| 2015 | Sistan and Baluchistan | Female | 6% (2.1-15.2) | 6.3% (2.2-15.3) | 18.2% (7-32.5) | 16.8% (6.2-31.7) |
|  |  | Male | 11% (3.9-20.6) | 11.1% (4-20.8) | 22.9% (10.5-37.2) | 23.4% (10.7-38.1) |
| 2015 | Tehran | Female | 5.9% (2-15.4) | 7% (2.6-16.6) | 17.6% (6.8-31.7) | 19.1% (7.3-34.2) |
|  |  | Male | 11.3% (4.2-20.8) | 12.1% (4.6-21.8) | 22.9% (10.4-37.1) | 26% (12.6-40.9) |
| 2015 | Yazd | Female | 6.7% (2.2-16.3) | 7.3% (2.6-17.1) | 17.2% (6.5-31.3) | 19.8% (7.2-34.6) |
|  |  | Male | 11.1% (4-20.6) | 12.1% (4.4-21.9) | 22% (9.6-36.2) | 26.6% (12.8-41.4) |
| 2015 | Zanjan | Female | 6.5% (2.2-16) | 6.8% (2.4-16.3) | 18.6% (7.2-32.6) | 20% (7.2-34.7) |
|  |  | Male | 11% (3.9-20.5) | 12.3% (4.3-22.1) | 23.7% (10.7-37.8) | 27% (13-41.8) |
| 2020 | Alborz | Female | 8.2% (2.8-17.7) | 9.1% (3.3-18.9) | 19.4% (7.9-33.7) | 22.4% (9-37.3) |
|  |  | Male | 12.7% (4.7-22.2) | 13.6% (5-23.5) | 26.5% (13-40.9) | 30% (15.2-45) |
| 2020 | Ardabil | Female | 7.5% (2.5-17) | 8.6% (2.9-18.4) | 21.4% (9-35.5) | 23.7% (9.3-38.5) |
|  |  | Male | 12.6% (4.8-22.2) | 13.7% (5.4-23.4) | 27.1% (13.4-41) | 31% (16.3-45.7) |
| 2020 | Azerbaijan, East | Female | 7.3% (2.5-16.8) | 7.6% (2.8-17.3) | 20.6% (8.4-34.8) | 22% (8.8-36.8) |
|  |  | Male | 12% (4.5-21.5) | 12.9% (5-22.6) | 26.3% (12.6-40.5) | 28.7% (14.5-43.6) |
| 2020 | Azerbaijan, West | Female | 6.5% (2.2-15.7) | 7.8% (3-17.4) | 18.6% (7.2-32.7) | 21.3% (8.4-36) |
|  |  | Male | 11.4% (4.1-20.8) | 12.8% (5-22.3) | 24.4% (11-38.5) | 28.8% (14.4-43.4) |
| 2020 | Bushehr | Female | 7.3% (2.4-16.9) | 7% (2.6-16.7) | 20.5% (8.3-34.8) | 21.6% (8.3-36.4) |
|  |  | Male | 12.9% (4.8-22.4) | 13.5% (5.1-23.3) | 25.7% (12.2-39.9) | 29.4% (15.1-44.2) |
| 2020 | Chahar Mahaal and Bakhtiari | Female | 7.1% (2.3-16.8) | 7.9% (2.8-17.6) | 19.6% (7.8-33.7) | 21.3% (8.1-36.1) |
|  |  | Male | 11.2% (3.9-20.8) | 12.2% (4.5-21.9) | 24.9% (11.7-39) | 26.8% (13.4-41.6) |
| 2020 | Fars | Female | 6.5% (2.3-16) | 7% (2.6-16.6) | 18.9% (7-32.9) | 21.1% (7.6-35.8) |
|  |  | Male | 11.3% (4.1-20.9) | 11.9% (4.6-21.7) | 24.3% (11.2-38.5) | 27.4% (13.4-42) |
| 2020 | Gilan | Female | 8.4% (2.8-17.9) | 9.2% (3.4-18.8) | 21.8% (9.3-36) | 25% (10.6-39.7) |
|  |  | Male | 12.8% (4.9-22.2) | 14.6% (5.7-24.3) | 26.6% (13-40.6) | 31.1% (16.3-45.7) |
| 2020 | Golestan | Female | 6.6% (2.1-16.1) | 7.1% (2.8-16.7) | 18.1% (6.9-31.9) | 21.2% (8.1-36) |
|  |  | Male | 11.5% (4.2-21) | 12.5% (4.9-22.1) | 23.8% (11.1-37.7) | 27.7% (13.9-42.5) |
| 2020 | Hamadan | Female | 6.5% (2.1-16) | 7.3% (2.6-17) | 18.3% (6.9-32.4) | 21.6% (8.2-36.4) |
|  |  | Male | 11.1% (4-20.7) | 11.5% (4.4-21.2) | 23% (10.2-37.2) | 26.9% (13-41.7) |
| 2020 | Hormozgan | Female | 6.9% (2.3-16.5) | 7.1% (2.5-16.9) | 19.9% (7.8-34.1) | 21.1% (7.5-35.9) |
|  |  | Male | 10.9% (3.8-20.5) | 11.8% (4.2-21.5) | 24.4% (11.3-38.5) | 26.2% (12.3-41.1) |
| 2020 | Ilam | Female | 6.4% (2-15.9) | 6.4% (2.1-16.2) | 20.2% (8.1-34.3) | 21.4% (7.4-36.3) |
|  |  | Male | 11% (3.7-20.5) | 11.5% (4.3-21.3) | 23.9% (11-37.8) | 27.1% (13-41.7) |
| 2020 | Isfahan | Female | 7.5% (2.5-17) | 7.9% (2.9-17.6) | 19.7% (7.9-33.7) | 20.5% (7.6-35.3) |
|  |  | Male | 11.4% (4.1-20.9) | 12.1% (4.5-21.9) | 25.1% (11.8-39.2) | 26.4% (12.6-41.3) |
| 2020 | Kerman | Female | 7.6% (2.5-17) | 7% (2.8-16.5) | 18.9% (7.1-33) | 18.4% (6.7-33.2) |
|  |  | Male | 11.8% (4.3-21.4) | 10.5% (4.1-20.3) | 23.7% (10.7-37.9) | 23.6% (11-38.2) |
| 2020 | Kermanshah | Female | 6.9% (2.3-16.4) | 8% (2.9-17.7) | 19% (7.2-33.2) | 21.6% (7.9-36.1) |
|  |  | Male | 11.7% (4.2-21.3) | 12.7% (4.9-22.4) | 24.2% (11.1-38.3) | 28.6% (14.3-43.3) |
| 2020 | Khorasan, North | Female | 6.5% (2.2-16) | 7% (2.4-16.7) | 20.5% (8.4-34.6) | 21.5% (8.1-36.3) |
|  |  | Male | 11.5% (4-21) | 12.4% (4.4-22.1) | 25.3% (11.8-39.3) | 28.7% (14.4-43.6) |
| 2020 | Khorasan, Razavi | Female | 6.9% (2.3-16.4) | 7.7% (2.8-17.5) | 18.1% (6.9-32.1) | 21.2% (7.8-36) |
|  |  | Male | 11.2% (4.1-20.6) | 12.7% (4.8-22.4) | 23.4% (10.8-37.4) | 27.6% (13.7-42.4) |
| 2020 | Khorasan, South | Female | 6.2% (2-15.7) | 6.2% (2.2-15.8) | 17.8% (6.6-31.8) | 19.3% (6.5-34.1) |
|  |  | Male | 10.9% (3.8-20.4) | 10.9% (4.1-20.6) | 22.6% (10-36.6) | 25.3% (12-40.2) |
| 2020 | Khuzestan | Female | 7.1% (2.4-16.6) | 8.3% (3.2-18.2) | 20.1% (8.2-34.2) | 22.6% (8.6-37.2) |
|  |  | Male | 12.3% (4.4-21.7) | 13.1% (5.1-22.8) | 26% (12.6-40) | 29.7% (15.2-44.3) |
| 2020 | Kohgiluyeh and Boyer-Ahmad | Female | 6.6% (2.3-16.1) | 7.9% (2.9-17.6) | 18.8% (7-33.1) | 21.4% (7.6-36.4) |
|  |  | Male | 11.4% (3.9-20.9) | 11.9% (4.6-21.6) | 24.1% (10.9-38.3) | 28.5% (14.1-43.4) |
| 2020 | Kurdistan | Female | 7% (2.4-16.5) | 8.3% (2.8-18.1) | 19.5% (7.5-33.6) | 22.5% (8.3-37.3) |
|  |  | Male | 11.7% (4-21.1) | 13.1% (4.8-22.8) | 24.7% (11.4-38.8) | 30.4% (15.9-45.1) |
| 2020 | Lorestan | Female | 6.9% (2.3-16.4) | 6.7% (2.6-16) | 20.5% (8.2-34.6) | 20.4% (7.1-35.2) |
|  |  | Male | 11.6% (4-21.1) | 11.3% (4.2-21) | 25.3% (11.9-39.6) | 26% (12.1-40.9) |
| 2020 | Markazi | Female | 7% (2.3-16.6) | 7.9% (2.8-17.6) | 19.2% (7.6-33.3) | 22.1% (8.7-36.9) |
|  |  | Male | 11.8% (4.2-21.2) | 12.9% (4.8-22.6) | 24.4% (11.4-38.6) | 28% (13.7-42.7) |
| 2020 | Mazandaran | Female | 8.1% (2.6-17.5) | 8.2% (3-18.1) | 20.7% (8.6-34.7) | 23.4% (9.5-38.1) |
|  |  | Male | 13% (5-22.6) | 13.5% (5.2-23.2) | 25.4% (11.9-39.7) | 29.5% (15.2-44.3) |
| 2020 | Qazvin | Female | 7.4% (2.4-17) | 7.5% (2.8-17.2) | 20% (8.1-34.1) | 21.8% (8.2-36.5) |
|  |  | Male | 12.1% (4.3-21.5) | 12.6% (4.8-22.3) | 25.6% (12.2-39.6) | 27.7% (13.3-42.5) |
| 2020 | Qom | Female | 7.2% (2.3-16.8) | 7.4% (2.8-17.1) | 20.4% (8.5-34.7) | 22.9% (8.8-37.6) |
|  |  | Male | 12.2% (4.5-21.8) | 12.6% (4.8-22.3) | 25% (12.1-39.2) | 29.1% (14.7-43.8) |
| 2020 | Semnan | Female | 7.5% (2.4-16.9) | 7.9% (2.8-17.7) | 20.3% (8.4-34.3) | 22.9% (9.2-37.4) |
|  |  | Male | 12.1% (4.5-21.7) | 12.5% (4.7-22.2) | 24.5% (11.5-38.6) | 27.6% (13.6-42.2) |
| 2020 | Sistan and Baluchistan | Female | 6.3% (2.1-15.6) | 6.5% (2.3-15.5) | 18.9% (7.3-33.1) | 18.1% (6.7-33.1) |
|  |  | Male | 11.3% (4.2-20.8) | 11.1% (4.3-20.7) | 23.5% (10.9-37.9) | 24.4% (11.4-39.2) |
| 2020 | Tehran | Female | 6.4% (2.2-15.9) | 7.5% (2.9-17.2) | 18.1% (7-32.2) | 21.8% (8.4-36.7) |
|  |  | Male | 12% (4.4-21.5) | 12.6% (4.8-22.3) | 23.6% (10.7-37.9) | 28% (13.8-42.8) |
| 2020 | Yazd | Female | 7.5% (2.5-17) | 7.8% (2.8-17.6) | 18.2% (7-32.5) | 22.4% (8.7-37) |
|  |  | Male | 11.9% (4.4-21.3) | 12.6% (4.7-22.5) | 23.3% (10.5-37.4) | 28.7% (14.2-43.4) |
| 2020 | Zanjan | Female | 7% (2.3-16.5) | 7% (2.4-16.8) | 19.4% (7.4-33.5) | 22.2% (8.4-37.1) |
|  |  | Male | 11.3% (4-20.9) | 12.5% (4.6-22.3) | 24.7% (11.3-38.7) | 28.9% (14.5-43.7) |
| 2025 | Alborz | Female | 8.6% (2.9-18.2) | 9.5% (3.5-19.2) | 19.5% (7.9-33.8) | 24.1% (9.7-39) |
|  |  | Male | 13.1% (4.9-22.7) | 14.2% (5.3-24) | 26.8% (13.2-41.2) | 31.9% (16.8-46.7) |
| 2025 | Ardabil | Female | 7.8% (2.6-17.4) | 9.1% (3.1-18.8) | 21.7% (8.9-35.9) | 26.1% (11.5-40.9) |
|  |  | Male | 13.1% (5-22.7) | 14.2% (5.7-24) | 27.7% (13.9-41.7) | 32.8% (18.2-47.7) |
| 2025 | Azerbaijan, East | Female | 7.6% (2.6-17.3) | 7.9% (3-17.6) | 20.9% (8.4-35) | 23.7% (9.6-38.6) |
|  |  | Male | 12.5% (4.7-22) | 13.2% (5.3-23) | 26.7% (12.8-41) | 30% (15.4-45) |
| 2025 | Azerbaijan, West | Female | 6.8% (2.4-16.3) | 8.1% (3.2-17.6) | 19.1% (7.4-33.1) | 22.7% (9.2-37.3) |
|  |  | Male | 11.8% (4.3-21.3) | 13.1% (5.3-22.9) | 25.1% (11.6-39.3) | 30% (15.2-44.7) |
| 2025 | Bushehr | Female | 8.3% (2.7-17.8) | 7.5% (2.8-17.1) | 20.8% (8.5-35) | 23.3% (9.1-38) |
|  |  | Male | 13.9% (5.4-23.3) | 14.2% (5.5-24) | 26% (12.4-40.4) | 31.5% (16.8-46.4) |
| 2025 | Chahar Mahaal and Bakhtiari | Female | 7.5% (2.4-17.1) | 8.1% (2.8-17.8) | 20.2% (8.1-34.4) | 23.1% (9.1-37.8) |
|  |  | Male | 11.5% (4.2-21.1) | 12.6% (4.8-22.3) | 25.6% (12.3-39.7) | 27.9% (14.1-42.7) |
| 2025 | Fars | Female | 6.9% (2.3-16.4) | 7.2% (2.8-16.8) | 19.2% (7.1-33) | 23.1% (8.3-37.8) |
|  |  | Male | 12% (4.6-21.6) | 12.3% (5-22.2) | 24.8% (11.5-38.9) | 28.8% (14.4-43.5) |
| 2025 | Gilan | Female | 8.8% (2.9-18.3) | 10% (3.7-19.7) | 22% (9.5-36.2) | 27.8% (13-42.5) |
|  |  | Male | 13.1% (5.1-22.5) | 15.1% (6.1-24.8) | 26.9% (13-41) | 33% (18-47.7) |
| 2025 | Golestan | Female | 7% (2.2-16.6) | 7.4% (2.9-17) | 18.7% (7.1-32.7) | 23% (9-37.9) |
|  |  | Male | 12% (4.5-21.6) | 12.6% (5.2-22.4) | 24.3% (11.4-38.5) | 28.9% (14.9-43.8) |
| 2025 | Hamadan | Female | 6.9% (2.1-16.4) | 7.6% (2.7-17.3) | 18.8% (7.1-32.9) | 23.6% (9.5-38.3) |
|  |  | Male | 11.3% (4.1-21) | 11.5% (4.5-21.2) | 23.8% (10.8-37.8) | 28.2% (13.9-43.1) |
| 2025 | Hormozgan | Female | 7.4% (2.3-17.1) | 7.6% (2.7-17.3) | 20.2% (8.1-34.4) | 23.1% (8.7-37.8) |
|  |  | Male | 11.3% (4-20.9) | 12% (4.3-21.7) | 24.9% (11.5-39) | 27.5% (13.2-42.2) |
| 2025 | Ilam | Female | 6.8% (2-16.4) | 6.8% (2.2-16.5) | 20.8% (8.3-34.7) | 23.6% (8.8-38.5) |
|  |  | Male | 11.5% (4-21.1) | 11.8% (4.5-21.7) | 24.3% (11.3-38.4) | 28% (13.8-43) |
| 2025 | Isfahan | Female | 7.8% (2.6-17.4) | 8.3% (3.1-18.1) | 19.7% (7.8-33.7) | 22.1% (8.6-36.9) |
|  |  | Male | 11.8% (4.3-21.4) | 12.5% (4.8-22.3) | 25.5% (12.2-39.7) | 27.5% (13.5-42.2) |
| 2025 | Kerman | Female | 7.9% (2.5-17.4) | 7.1% (2.8-16.5) | 19.4% (7.3-33.4) | 19.1% (7.1-34) |
|  |  | Male | 11.9% (4.5-21.6) | 10.2% (4.2-20) | 24.2% (11.2-38.2) | 23.7% (11.1-38.5) |
| 2025 | Kermanshah | Female | 6.9% (2.4-16.5) | 8.1% (3.1-17.8) | 19.4% (7.2-33.3) | 22.9% (8.4-37.3) |
|  |  | Male | 12.1% (4.3-21.5) | 12.9% (5-22.7) | 24.8% (11.4-38.9) | 29.9% (15.2-44.6) |
| 2025 | Khorasan, North | Female | 7% (2.3-16.6) | 7.5% (2.5-17.2) | 21.1% (8.7-35.2) | 23.5% (9.3-38.5) |
|  |  | Male | 12.1% (4.3-21.7) | 13% (4.8-22.6) | 25.8% (12.3-39.9) | 30.9% (16.1-45.6) |
| 2025 | Khorasan, Razavi | Female | 7.2% (2.4-16.7) | 8.1% (2.9-17.7) | 18.5% (7.1-32.7) | 22.5% (8.5-37.2) |
|  |  | Male | 11.6% (4.3-21.1) | 12.9% (5-22.7) | 23.9% (11.1-38.1) | 28.5% (14.6-43.2) |
| 2025 | Khorasan, South | Female | 6.5% (2-16.1) | 6.6% (2.2-16) | 18.5% (6.8-32.5) | 21.1% (7.6-35.9) |
|  |  | Male | 11.3% (4.1-20.9) | 11.2% (4.4-20.9) | 23.6% (10.6-37.6) | 27.1% (13.4-42) |
| 2025 | Khuzestan | Female | 7.4% (2.5-17) | 8.6% (3.3-18.4) | 20.4% (8.2-34.5) | 24% (9.4-38.6) |
|  |  | Male | 12.5% (4.6-22.1) | 13.2% (5.3-23) | 26.6% (13-40.8) | 31.1% (16.1-45.7) |
| 2025 | Kohgiluyeh and Boyer-Ahmad | Female | 6.8% (2.3-16.4) | 8.5% (3.2-18.3) | 19.3% (7.2-33.5) | 23.6% (9-38.6) |
|  |  | Male | 11.7% (4.1-21.3) | 12.4% (4.9-22.1) | 25% (11.4-39.1) | 30.1% (15.3-45) |
| 2025 | Kurdistan | Female | 7.3% (2.5-16.8) | 8.7% (3.1-18.4) | 20% (7.7-34) | 24.4% (9.5-39.2) |
|  |  | Male | 11.9% (4.3-21.6) | 13.5% (5.1-23.3) | 25.4% (12-39.4) | 32.1% (17.4-46.8) |
| 2025 | Lorestan | Female | 7.3% (2.3-16.8) | 6.9% (2.6-16.5) | 20.9% (8.3-35) | 22% (7.7-36.9) |
|  |  | Male | 11.9% (4.2-21.4) | 11.1% (4.5-20.8) | 25.9% (12.2-39.9) | 26.9% (12.6-41.8) |
| 2025 | Markazi | Female | 7.8% (2.5-17.3) | 8.6% (3.1-18.4) | 19.5% (7.7-33.8) | 24.7% (10.2-39.6) |
|  |  | Male | 12.5% (4.8-22) | 13.7% (5.4-23.6) | 25.1% (11.8-39.2) | 29.9% (15.2-44.7) |
| 2025 | Mazandaran | Female | 8.6% (2.7-18.1) | 8.9% (3.2-18.7) | 21.1% (8.8-35.1) | 25.9% (11.5-40.5) |
|  |  | Male | 13.4% (5.2-23) | 13.7% (5.5-23.6) | 26.1% (12.5-40.2) | 30.9% (16.3-45.7) |
| 2025 | Qazvin | Female | 7.7% (2.5-17.3) | 7.9% (3.1-17.8) | 20.2% (8.1-34.4) | 23.7% (9.2-38.5) |
|  |  | Male | 12.3% (4.3-21.8) | 13% (5-22.7) | 26% (12.4-40.1) | 28.9% (14.3-43.6) |
| 2025 | Qom | Female | 7.9% (2.6-17.6) | 8% (2.9-17.7) | 20.7% (8.6-35) | 24.9% (10.4-39.6) |
|  |  | Male | 12.6% (4.6-22.2) | 13.1% (5-22.7) | 25.7% (12.5-39.8) | 30.6% (15.9-45.3) |
| 2025 | Semnan | Female | 8% (2.6-17.5) | 8.4% (3-18.2) | 20.8% (8.8-34.8) | 25.4% (11.1-40.1) |
|  |  | Male | 12.5% (4.8-22) | 12.8% (4.9-22.6) | 25.2% (11.7-39.2) | 28.9% (14.5-43.7) |
| 2025 | Sistan and Baluchistan | Female | 6.4% (2.2-15.8) | 6.8% (2.5-15.7) | 19% (7.3-33.4) | 19% (7.2-33.8) |
|  |  | Male | 11.4% (4.1-21) | 11% (4.3-20.6) | 23.9% (11.1-38.2) | 25% (11.7-39.9) |
| 2025 | Tehran | Female | 6.6% (2.2-16.1) | 7.6% (2.9-17.3) | 18.3% (7-32.3) | 22.8% (8.9-37.8) |
|  |  | Male | 12.2% (4.4-21.7) | 12.7% (4.8-22.4) | 23.9% (11-38.2) | 28.5% (14.3-43.3) |
| 2025 | Yazd | Female | 8.3% (2.6-17.8) | 8.6% (3.1-18.4) | 19% (7.4-33.1) | 24.9% (10.1-39.7) |
|  |  | Male | 12.4% (4.8-22) | 12.9% (4.9-22.6) | 24.3% (11.1-38.5) | 30.7% (15.9-45.3) |
| 2025 | Zanjan | Female | 7.3% (2.4-16.8) | 7.5% (2.6-17.3) | 19.8% (7.7-33.9) | 24.6% (9.8-39.4) |
|  |  | Male | 11.8% (4.2-21.4) | 13% (4.8-22.7) | 25.4% (11.6-39.5) | 30.9% (16.1-45.7) |
| 2030 | Alborz | Female | 9.1% (3-18.8) | 10% (3.6-19.6) | 19.8% (8-34.2) | 25.1% (10.5-40) |
|  |  | Male | 13.1% (5.1-22.8) | 14.4% (5.4-24.3) | 27.2% (13.5-41.3) | 33.5% (18.2-48.3) |
| 2030 | Ardabil | Female | 8.9% (2.8-18.6) | 9.8% (3.2-19.5) | 21.9% (8.8-36.2) | 28.6% (13.8-43.3) |
|  |  | Male | 14% (5.8-23.3) | 15% (6.4-24.8) | 28.3% (14.4-42.5) | 34.8% (19.9-49.4) |
| 2030 | Azerbaijan, East | Female | 8.1% (2.4-17.6) | 8.8% (3.2-18.6) | 21% (8.3-35.2) | 25.4% (10.8-40.4) |
|  |  | Male | 11.7% (4.6-21.2) | 12.9% (5.3-22.7) | 27.2% (13.2-41.2) | 31.5% (16.8-46.2) |
| 2030 | Azerbaijan, West | Female | 7.2% (2.4-16.6) | 8.6% (3.3-18.1) | 19.5% (7.6-33.5) | 24.4% (10.2-39) |
|  |  | Male | 11.8% (4.6-21.1) | 13.1% (5.7-22.7) | 26.1% (12.1-40.4) | 31.7% (16.7-46.5) |
| 2030 | Bushehr | Female | 8.7% (2.6-18.2) | 7.8% (2.8-17.7) | 20.9% (8.4-35.1) | 24.8% (10.3-39.6) |
|  |  | Male | 13.7% (5.4-23.3) | 14.4% (5.9-24) | 26.3% (12.8-40.5) | 33.3% (18.6-48.1) |
| 2030 | Chahar Mahaal and Bakhtiari | Female | 7.8% (2.6-17.3) | 8.2% (2.9-18) | 20.7% (8.6-34.7) | 25% (10.7-39.7) |
|  |  | Male | 12.1% (4.3-21.7) | 13.1% (5-22.8) | 26.4% (12.9-40.6) | 29.3% (15-44.1) |
| 2030 | Fars | Female | 7.1% (2.5-16.6) | 7.6% (3.1-17.3) | 19.3% (6.9-33.5) | 24.8% (10-39.3) |
|  |  | Male | 12.8% (4.7-22.4) | 12.8% (5.1-22.8) | 25.4% (11.8-39.7) | 30.2% (15.4-44.9) |
| 2030 | Gilan | Female | 9.1% (3.2-18.8) | 10.7% (4.3-20.5) | 22.3% (9.6-36.2) | 30.2% (15.6-45) |
|  |  | Male | 13.5% (5.5-22.8) | 15.8% (6.6-25.6) | 27.1% (13.2-41.2) | 34.5% (19.7-49) |
| 2030 | Golestan | Female | 7.2% (2.4-16.8) | 7.8% (3.1-16.9) | 19.2% (7.3-33.4) | 24.7% (10.1-39.8) |
|  |  | Male | 12.8% (4.7-22.4) | 13.3% (5.3-23.1) | 25% (11.7-39.1) | 30% (15.7-45) |
| 2030 | Hamadan | Female | 6.9% (2.3-16.5) | 7.7% (2.9-17.4) | 19.3% (7.1-33.4) | 25.5% (10.9-40.4) |
|  |  | Male | 12.2% (4.4-21.7) | 12% (4.7-21.6) | 24.9% (11.4-39) | 29.7% (15-44.5) |
| 2030 | Hormozgan | Female | 7.8% (2.6-17.3) | 8% (3-17.9) | 20.5% (8.2-34.5) | 24.9% (10.1-39.6) |
|  |  | Male | 11.7% (4.2-21.3) | 12.3% (4.5-22.2) | 25.3% (11.6-39.3) | 28.6% (14.5-43.3) |
| 2030 | Ilam | Female | 7.5% (2.1-17.1) | 7.3% (2.4-17.2) | 21.3% (8.3-35.4) | 25.9% (11.1-40.9) |
|  |  | Male | 11.8% (4.4-21.5) | 12.1% (5.1-22) | 24.8% (11.4-38.8) | 29.6% (14.8-44.1) |
| 2030 | Isfahan | Female | 8.4% (2.4-18) | 8.5% (2.9-18.3) | 19.8% (7.7-33.9) | 23.5% (9.4-38.2) |
|  |  | Male | 12.4% (4.9-21.9) | 12.8% (5.2-22.6) | 26% (12.4-40.5) | 28.6% (14.4-43.3) |
| 2030 | Kerman | Female | 8.1% (2.8-17.6) | 6.9% (3.1-16.3) | 19.7% (7.4-34) | 19.9% (7.5-34.7) |
|  |  | Male | 12.2% (4.6-21.9) | 9.9% (4.4-19.7) | 24.7% (11.3-38.8) | 24% (11.3-38.6) |
| 2030 | Kermanshah | Female | 7.1% (2.3-16.4) | 8.3% (3.1-17.9) | 19.6% (7.1-33.6) | 24.2% (9.6-38.9) |
|  |  | Male | 12.3% (4.3-21.7) | 13.2% (5.1-22.9) | 25.5% (11.7-39.5) | 31% (16.2-45.4) |
| 2030 | Khorasan, North | Female | 7.5% (2.4-17.1) | 7.7% (2.8-17.6) | 21.5% (8.8-35.6) | 26% (11.3-40.8) |
|  |  | Male | 12.8% (4.6-22.4) | 13.7% (5.2-23.5) | 26.4% (12.7-40.4) | 32.9% (17.9-48) |
| 2030 | Khorasan, Razavi | Female | 7.9% (2.5-17.4) | 8.7% (3.1-18.3) | 18.9% (7.3-33.2) | 23.8% (9.8-38.5) |
|  |  | Male | 11.8% (4.5-21.5) | 13% (5.5-22.8) | 24.5% (11.5-38.8) | 29.4% (15.4-43.9) |
| 2030 | Khorasan, South | Female | 6.6% (2.3-16.3) | 6.7% (2.6-16.5) | 19.2% (7.2-33.1) | 23% (8.8-38) |
|  |  | Male | 11.7% (4.3-21.3) | 11.5% (4.7-21.1) | 24.4% (11.5-38.8) | 28.8% (14.7-43.7) |
| 2030 | Khuzestan | Female | 7.6% (2.6-17) | 8.6% (3.5-18.3) | 20.7% (8.4-35.1) | 25.5% (10.6-40.2) |
|  |  | Male | 13.3% (4.8-22.4) | 13.9% (5.4-23.5) | 27.2% (13.6-41.5) | 32.4% (17.7-47.2) |
| 2030 | Kohgiluyeh and Boyer-Ahmad | Female | 6.7% (2-16.3) | 8.4% (2.9-18.3) | 19.9% (7.2-33.9) | 25.7% (11.2-40.5) |
|  |  | Male | 12% (4.6-21.8) | 13.8% (6-23.6) | 25.8% (12-39.8) | 32.1% (17.5-47.3) |
| 2030 | Kurdistan | Female | 7.4% (2.4-16.8) | 9% (3.3-18.7) | 20.4% (7.9-34.6) | 26.5% (11.3-41.4) |
|  |  | Male | 12.4% (4.3-22) | 14.1% (5.6-23.9) | 26.2% (12.5-40.4) | 34% (19.4-48.8) |
| 2030 | Lorestan | Female | 7.5% (2.5-17.2) | 6.9% (3-16.5) | 21.3% (8.3-35.5) | 23.9% (9.2-38.8) |
|  |  | Male | 12.3% (4.6-22) | 11.4% (4.7-21.2) | 26.5% (12.8-40.7) | 28% (13.5-43) |
| 2030 | Markazi | Female | 8.2% (2.6-17.9) | 9.3% (3.5-19) | 19.9% (7.8-34.1) | 27.1% (12.3-41.7) |
|  |  | Male | 13.2% (5-22.7) | 14.7% (5.9-24.4) | 25.9% (12.4-39.9) | 31.7% (17-46.5) |
| 2030 | Mazandaran | Female | 9.3% (2.8-18.9) | 9.6% (3.5-19.4) | 21.5% (8.9-35.7) | 28.2% (13.4-43) |
|  |  | Male | 13.5% (5.5-22.8) | 13.8% (5.9-23.8) | 26.9% (12.9-40.6) | 32.2% (17.5-46.9) |
| 2030 | Qazvin | Female | 7.7% (2.3-17.5) | 8.2% (3.1-17.9) | 20.3% (8-34.4) | 25.1% (10.5-39.7) |
|  |  | Male | 12.5% (4.3-22.2) | 13.4% (5.1-23.1) | 26.5% (13-40.6) | 30.2% (15.1-44.5) |
| 2030 | Qom | Female | 8.4% (2.5-18.2) | 8.6% (2.8-17.9) | 21.1% (8.6-35.3) | 26.6% (11.8-41.5) |
|  |  | Male | 12.6% (4.9-21.9) | 12.7% (5.1-22.4) | 26.5% (12.9-40.8) | 31.7% (16.8-46.4) |
| 2030 | Semnan | Female | 7.9% (2.8-17.5) | 8.3% (3.2-18.1) | 21.3% (8.9-35.4) | 27.6% (13-42.2) |
|  |  | Male | 13.4% (4.9-22.8) | 13.5% (5.1-23.2) | 25.9% (12.3-39.9) | 30.1% (15.9-44.9) |
| 2030 | Sistan and Baluchistan | Female | 6.7% (2.1-16.4) | 7% (2.5-16.1) | 19.4% (7.3-33.6) | 20% (7.7-34.6) |
|  |  | Male | 11.2% (4.4-20.8) | 10.6% (4.4-20.4) | 24.3% (11.2-38.7) | 25.4% (11.9-40.1) |
| 2030 | Tehran | Female | 6.8% (2.3-16.4) | 7.7% (2.9-17.4) | 18.5% (7-32.5) | 23.6% (9.1-38.3) |
|  |  | Male | 12% (4.6-21.6) | 12.5% (4.9-22.2) | 24.5% (11.2-38.8) | 28.8% (14.5-43.7) |
| 2030 | Yazd | Female | 8.6% (2.9-18.2) | 9.1% (3.4-18.9) | 19.6% (7.6-34) | 27.2% (12.6-42) |
|  |  | Male | 12.9% (4.8-22.4) | 13.3% (5-23) | 25.5% (11.8-39.9) | 32.4% (17.7-47.2) |
| 2030 | Zanjan | Female | 7.5% (2.4-17.2) | 8.1% (2.7-17.8) | 20.3% (7.7-34.4) | 26.8% (12.1-41.9) |
|  |  | Male | 12.4% (4.3-22.1) | 13.5% (5-23.2) | 26.1% (12.1-40.3) | 32.8% (18.2-47.6) |
